# The paradox of autophagy in Tuberous Sclerosis Complex

**DOI:** 10.1590/1678-4685-GMB-2020-0014

**Published:** 2021-04-05

**Authors:** Larissa Brussa Reis, Eduardo C. Filippi-Chiela, Patricia Ashton-Prolla, Fernanda Visioli, Clévia Rosset

**Affiliations:** 1 Hospital de Clínicas de Porto Alegre (HCPA), Serviço de Pesquisa Experimental, Laboratório de Medicina Genômica, Porto Alegre, RS, Brazil.; 2 Universidade Federal do Rio Grande do Sul (UFRGS), Programa de Pós-Graduação em Genética e Biologia Molecular, Porto Alegre, RS, Brazil.; 3 Universidade Federal do Rio Grande do Sul (UFRGS), Instituto de Ciências Básicas da Saúde, Departamento de Ciências Morfológicas, Porto Alegre, RS, Brazil.; 4 Hospital de Clínicas de Porto Alegre (HCPA), Serviço de Genética Médica, Porto Alegre, RS, Brazil.; 5 Universidade Federal do Rio Grande do Sul, Faculdade de Odontologia, Departamento de Patologia Oral, Porto Alegre, RS, Brazil.

**Keywords:** Autophagy, mTOR signaling, Tuberous Sclerosis Complex

## Abstract

Tuberous sclerosis complex (TSC) is an autosomal dominant genetic disorder caused by germline mutations in *TSC1* or *TSC2* genes, which leads to the hyperactivation of the mTORC1 pathway, an important negative regulator of autophagy. This leads to the development of hamartomas in multiple organs. The variability in symptoms presents a challenge for the development of completely effective treatments for TSC. One option is the treatment with mTORC1 inhibitors, which are targeted to block cell growth and restore autophagy. However, the therapeutic effect of rapamycin seems to be more efficient in the early stages of hamartoma development, an effect that seems to be associated with the paradoxical role of autophagy in tumor establishment. Under normal conditions, autophagy is directly inhibited by mTORC1. In situations of bioenergetics stress, mTORC1 releases the Ulk1 complex and initiates the autophagy process. In this way, autophagy promotes the survival of established tumors by supplying metabolic precursors during nutrient deprivation; paradoxically, excessive autophagy has been associated with cell death in some situations. In spite of its paradoxical role, autophagy is an alternative therapeutic strategy that could be explored in TSC. This review compiles the findings related to autophagy and the new therapeutic strategies targeting this pathway in TSC.

## Tuberous Sclerosis Complex: Epidemiology and manifestations

Tuberous sclerosis complex (TSC - ORPHA: 805) has an extremely variable disease profile that has the potential to affect any organ of the body and is characterized mainly by the development of hamartomas in the skin, brain, kidneys, lungs, and heart (Northrup and Krueger, 2013). Hamartomas are defined as benign proliferation of mature tissues that grow aberrantly and often with disorganized architecture, which may occur in any body site (Tjarks et al., 2019). The incidence of TSC is approximately 1 in 6,000 live births, with prevalence in the population of 1 in 20,000 individuals, with no sexual or racial predilection (Sahin et al., 2016). The clinical manifestations of TSC vary widely. The involvement of the skin and mucous membranes is marked, with alterations identified in approximately 70% of the cases (Henske et al., 2016). The onset of symptoms may happen soon after birth and to four years of age. Congenital hypopigmented macules (HM) in the skin, the first visible symptom, usually precedes epilepsy. In addition to HM, there are other similar skin features, such as facial angiofibromas and subungual fibromas (Sahin *et al.*, 2016). Regarding the central nervous system (CNS), epilepsy is the most common symptom, affecting up to 90% of TSC patients, of whom two-thirds show symptoms before the second year of life. Cognitive deficit affects approximately 60 to 70% of patients and is often linked to seizures (Northrup and Krueger, 2013). The severity of seizures and other symptoms of the CNS depend on the presence, location, number and size of the brain hamartomas - called tubers. The three major intracranial lesions associated with TSC are cortical tubers, subependymal nodules and subependymal giant cell astrocytomas (SEGAs) (Shepherd et al., 1991), which are found in 5 to 20% of patients. SEGAs constitute more than 90% of the intracranial tumors associated with the disease and are responsible for 25% of the mortality attributed to TSC (Nabbout et al., 1999; Adriaensen *et al.*, 2009). Regarding other sites that may be affected in TSC, cardiac rhabdomyomas (benign tumors of the heart) are observed in up to 50% of patients and it can be detected in the fetus as early as 22 weeks gestation. Tumors may be single or multiple, may reach 3-25 mm, and are usually located in the cardiac ventricles along the septum. There is a strong association of germline *TSC1* or *TSC2* mutations and cardiac rhabdomyomas, with a mutation being identified in up to 93% of affected patients, in both patients with single and multiple tumors. Tumors may compromise ventricular functions, resulting in obstruction of blood flow and very rarely lead to arrhythmia, valvular defects, or cardiac failure. In most cases, however, rhabdomyomas are not hemodynamically relevant and do not increase in size, in fact, they tend to involute and disappear after the age of 3 (Tworetzky et al., 2003, Altmann et al. 2019; Uysal and Sahin, 2020). Renal abnormalities are another important morbidity factor in TSC and are considered the second leading cause of premature death. More than 70% of TSC patients develop multiple and bilateral angiomyolipomas, which usually occur in the kidneys and reach large dimensions (Henske *et al.*, 2016). Although benign in nature these tumors can rupture and bleed and ultimately cause renal failure. Algorithms for the management of AML have been developed and treatment intervention is recommended for TSC-associated AML >3cm in diameter. Therefore, they should be followed closely for timely intervention. Renal cysts are also commonly identified, with and without angiomyolipomas and can result in hypertension or kidney failure (Hatano and Egawa, 2020; Uysal and Sahin, 2020). The main pulmonary manifestation of TSC is lymphangioleiomyomatosis (LAM), which is associated with the infiltration of smooth muscle cells in all lung structures (Adriaensen *et al.*, 2011). This manifestation may occur later, with patients presenting symptoms in the third to fourth decade of life. LAM-compatible cystic lung parenchymal abnormalities are observed in 30 to 40% of women and in 10 to 12% of men with TSC, but symptomatic changes are quite rare in men (Adriaensen *et al.*, 2011; Cudzilo et al., 2013). Some reports consider that LAM is almost exclusively observed in adult women with TSC, suggesting that it is an estrogen-dependent phenotype, which has been actually demonstrated in animal studies. (Uysal and Sahin, 2020; Xu et al., 2020). In a seminal clinical trial led by McCormack et al. (2011), which included 89 patients with LAM, Sirolimus stabilized lung function, reduced serum VEGF-D levels, and was associated with a reduction in symptoms and improvement in quality of life. This and other studies led to the FDA approval of Rapamycin (Sirolimus) for lymphangioleiomyomatosis treatment in 2015.

The criteria for clinical and genetic diagnoses of TSC are shown in Table S1 (based on Refs. Northrup and Krueger, 2013; Sahin et al., 2016). At least 60% of TSC patients have no family history of the disease (Henske et al., 2016).

## Genetics and metabolic Pathways involved in Tuberous Sclerosis Complex

TSC is an autosomal dominant disease caused by mutations that inactivate one of the two tumor-suppressor genes *TSC1* (OMIM 605284) or *TSC2* (OMIM 191092). The *TSC1* gene, located on chromosome 9q34, spans approximately 53 kb of genomic DNA, with 23 exons coding for the protein hamartin, a hydrophilic protein of 1164 amino acids and 130 kDa that interacts and stabilizes the GTPase activating protein tuberin, which is encoded by the *TSC2* gene. The *TSC2* gene, located on chromosome 16p13.3, comprises approximately 40 kb of genomic DNA and has 41 exons that generate a protein of 1807 amino acids and 200 kDa, possibly acting as a chaperone for hamartin. These two proteins together, along with TBC1D7 (Tre2-Bub2-Cdc16-1 domain family member 7) (Dibble and Manning, 2013), form the hamartin-tuberin complex, also called the TSC1/TSC2 complex or TSC complex. This complex acts as a GTPase activating protein (GAP) to inhibit the Ras-related small GTPase protein RHEB (Ras homologue enriched in brain) (Menon et al., 2014), which, in turn, regulates activation of the rapamycin target complex 1 in mammals (mTORC1) (Northrup et al., 2020). TSC complex is also required for proper activation of a second complex, called mTORC2 (Huang et al., 2008).

## mTOR complexes

The mTORC1 complex is an important regulator of cell growth, proliferation and translation of mRNAs and is sensitive to growth factors, nutrients and the energy status of the cells. This complex is formed by different subunits. The major catalytic subunit is mTOR, a highly conserved protein kinase that regulates cell cycle progression in vertebrates (Hay and Sonenberg, 2004). mTOR is associated with other proteins, such as the regulator-associated protein of mammalian target of rapamycin called Raptor, mLST8 (mammalian lethal with sec-13 protein 8, also known as GβL), and the recently identified subunits PRAS40 (proline-rich Akt substrate of 40 kDa) and DEPTOR (DEP domain-containing mTOR-interacting protein). mTORC2 is structurally and functionally distinct from mTORC1. Although mTORC2 is conserved, as mTORC1, it has a distinct catalytic subunit, called Rictor. mTORC2 controls the actin cytoskeleton and it is rapamycin insensitive, whereas mTORC1 is rapamycin sensitive (Wullschleger et al., 2006).

## Response to growth factor, nutrients and energy status

**Figure 1 -:**
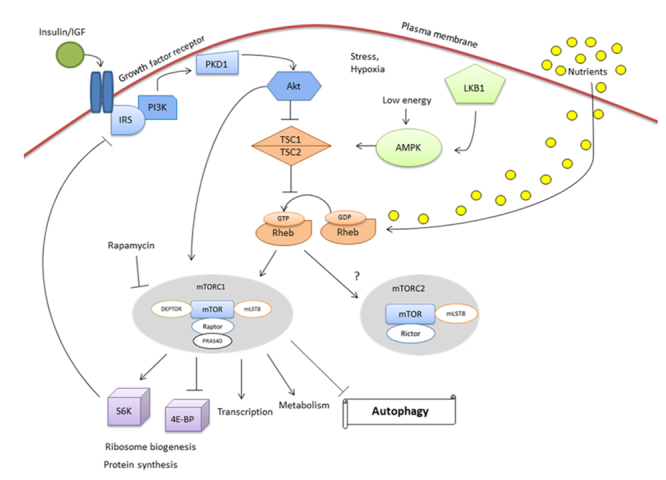
Signaling of TSC and mTOR complexes. TSC complex negatively regulates mTOR signaling. In response to the presence of insulin, tuberin is phosphorylated and functionally inactivated by Akt, allowing mTORC1 to perform its functions. Nutrients regulate mTORC1 signaling by the inhibition of the TSC complex. mTORC1 complex is sensitive to the energetic status of the cell through AMPK, which is activated in response to low energy in the cell level of AMP/ATP. Activated AMPK leads to inhibition of mTORC1. It is not completely clear which factors act upstream of mTORC2. Growth factors and amino acids regulate actin polymerization, suggesting that they may also regulate this complex through TSC and Rheb. Once activated, mTORC2 increases the cascade of mTOR signaling.

mTORC1 responds to growth factors via the phosphatidylinositol-4,5-bisphosphate 3-kinase pathway (PI3K). In response to the presence of insulin, tuberin is phosphorylated and functionally inactivated by Protein kinase B (Akt). The phosphorylation impairs the ability of the TSC complex to exert its GTPase activity that converts Ras homolog enriched in brain (RHEB) GTP-binding to RHEB-GDP. The accumulation of RHEB-GTP potentially activates mTORC1, which phosphorylates and inhibits the 4E-BP1 substrate and activates the substrates ribosomal protein S6 kinase beta-1 (S6K1) and beta-2 (S6K2) (Wullschleger et al., 2006), which promotes protein translation. In a negative feedback loop, mTORC1 and S6K1 directly phosphorylate insulin receptor 1 (IRS1) and block the signal transduction from insulin or insulin-like growth factor 1 (IGF-1) to PI3K (Huang and Manning, 2008) (Figure 1). Nutrients, especially amino acids, also regulate mTORC1 signaling. Nutrients inhibit the TSC complex by phosphorylation of S6K1 and eukaryotic initiation factor 4E-binding protein 1 (4E-BP1) (Gao et al., 2002). Alternatively, the nutrients can regulate the TSC complex independently of mTORC1 by inducing RHEB stimulation, which causes the rapid dephosphorylation of the same targets (Saucedo et al., 2003) (Figure 1). In relation to the high energy levels required for its activation, the mTORC1 complex is sensitive to the energy status of the cell as mediated through AMP-activated protein kinase (AMPK), which is activated in response to low energy at the cellular level of AMP/ATP. Activated AMPK is able to phosphorylate tuberin directly and thus increases its GTPase activity, leading to inhibition of mTORC1 (Inoki et al., 2003). It has been suggested that the tumor suppressor LKB1 is linked to the TSC-mTORC1 signaling pathway. Upon energy deprivation and in conjunction with AMP, LKB1 activates AMPK, which in turn phosphorylates and activates TSC2, resulting in the inhibition of mTORC1 (Wullschleger *et al.*, 2006). The factors that act upstream of mTORC2 are not well known. Growth factors and amino acids regulate actin polymerization, suggesting that they may also regulate the mTORC2 complex through TSC and RHEB (Jacinto et al., 2004). Once activated, mTORC2 phosphorylates the downstream target Akt to increase the mTOR signaling cascade (Xie and Proud, 2014) (Figure 1).

mTORC1 is a major negative regulator of autophagosome formation (Hung et al., 2012). In yeast and mammals, the activation of the Atg1/ULK1 complex, formed by ULK1-ATG13-FIP200, is essential for the canonical formation of autophagosomes (Hosokawa et al., 2009). The activity of this complex is negatively regulated by mTORC1. When nutrients are available, mTORC1 is activated and the ULK1 complex is inactivated through the phosphorylation of ULK1 and ATG13, thus suppressing autophagy; on the other hand, in this context, ribosomal biogenesis and protein synthesis are stimulated. Conversely, under nutrient limitations, mTORC1 is inactivated, which enables the ULK1 complex to be activated, which triggers the formation of autophagosomes. In parallel to autophagy induction, the inactivation of mTORC inhibits cell growth (Balgi et al., 2009).

## Therapies targeting mTOR complexes in TSC

Some therapies directed to the inhibition of mTOR complexes in the TSC context have been proposed. First-generation mTOR inhibitors consist of rapamycin (MacKeigan and Krueger, 2015). Rapamycin is an immunosuppressor that forms an inhibitory complex with the immunophilin FKBP12, which then binds and inhibits the ability of mTORC1 to phosphorylate downstream substrates such as S6Ks and 4EBPs. Since 2001 it has been approved for a large number of therapeutic uses in the United States and demonstrates beneficial results in reducing the volume of SEGAs (Franz et al., 2006) and renal angiomyolipomas (Cabrera-López et al., 2012), reducing the number of facial angiofibromas (Koenig et al., 2018), and controlling epileptic seizures (Zou et al., 2014). Rapamycin analogs (also called rapalogs) are being increasingly used not only for hamartomatous and oncological manifestations of TSC but also as adjunct therapies for epilepsy and intellectual disability among others desabilities (Franz and Capal, 2017). More recently, second-generation mTOR inhibitors have been developed. In contrast to rapamycin and its analogs, these molecules do not target FKBP12 but inhibit both mTORC1 and mTORC2 directly by blocking their catalytic sites through competition with ATP. These new agents, called Everolimus and Sirolimus are potent inhibitors of cell proliferation and may have therapeutic benefits in TSC. Some studies indicate that inhibition of mTORC1 and mTORC2 together could be more appropriate than the use of rapalogs alone, since the dual approach could prevent activation of mTORC2 that may result from the inhibition of mTORC1 alone (Julien et al., 2010). Knowing that mTORC2 phosphorylates Akt to activate it and thus promotes cell survival through many downstream signaling targets, the loss of mTORC2-mediated Akt activation in cells without a functional TSC complex may effectively suppress apoptosis-inducing stimuli. For these reasons, many investigators prefer to consider both the aberrant activation of mTORC1 and the inactivation of mTORC2 when developing therapeutic strategies for TSC (Huang et al., 2008). Important clinical trials developed in recent years that have changed systemic treatments in TSC patients include EXIST-1 trial, completed in 2016 that demonstrated the efficacy and safety of Everolimus in SEGAs (Kingswood et al., 2014; Franz *et al.*, 2016) and EXIST-2 trial that demonstrated the benefit of mTOR inhibitors for renal angiomyolipomas (AMLs) and resulted in Everolimus approval by the FDA for asymptomatic and growing renal AMLs larger than 3 cm (Bissler et al. 2013). Some years later, Bissler *et al.* (2017) treated 112 patients with Everolimus in an extension phase of the EXIST-2 study for an average of 46.9 months and observed that 58% achieved some AML response, the majority being reduction in renal lesion volumes with no AML-related bleeding or nephrectomies being reported. The most common adverse events suspected to be treatment-related were stomatitis, hypercholesterolemia, acne, aphthous stomatitis and nasopharyngitis but less than 10% of patients withdrew treatment due to an adverse event. In addition Bissler *et al.* (2018) reported that in a series of 33 patients with TSC treated with Everolimus for SEGAs, renal angiomyolipoma response was achieved by 75.8% of patients with sustained reductions in tumor volume over nearly 4 years of treatment, reaching ≥50% in most (≥80%) patients. Beneficial effects of Everolimus on autism and attention-deficit/hyperactivity disorder symptoms have been reported also (Kilincaslan et al., 2017). Regarding tests involving Sirolimus, MILES trial included 89 patients with LAM and reported stabilized lung function, reduced serum VEGF-D levels that were associated with a reduction in symptoms and improvement in the quality of life for TSC patients with LAM (McCormack et al. 2011). These results led to the FDA approval of Sirolimus in TSC-associated LAM in 2015. It is important to consider that both mTORC1 and mTORC2 inhibitors cause a variety of side effects that can lead to life-threatening outcomes, including sepsis and death. In addition, another point that requires further research relates to the long-lasting effects of these treatments, particularly in the context of the high incidence of lifetime clinical features and the adult- onset symptoms of TSC patients (Trelinska et al., 2015). For most TSC-related hamartomas, lifelong treatment will likely be mandatory, since several lesions may regrow and complications of these tumors may recur when medications are discontinued. One of the challenges in TSC treatment is that different clinical manifestations of the disease may require different therapeutic interventions. A key point is timing of mTOR inhibition for each symptom and what other pharmacologic and nonpharmacologic interventions could be used in combination. The main mTOR inhibitors tested in patients with TSC-related manifestations are summarized in Table S2.

## Basics of autophagy

Autophagy is a physiological and well-regulated cellular mechanism that degrades dysfunctional or unnecessary factors, enabling the recycling of cellular components and the maintenance of energy and structural homeostasis. It occurs through the capture of cytoplasmic fractions and organelles by autophagosome, which are digested in lysosomes. The final products of lysosomal digestion are returned to the cytosol. They are then used in cellular metabolism to generate energy and to build new proteins, organelles and membrane components. When nutrients are restricted, autophagy is increased to ensure an internal source of nutrients for energy supply and, consequently, for cell survival. Therefore, it is a powerful mechanism to promote metabolic homeostasis at both the cellular and organism levels (Rabinowitz and White, 2010).

Autophagy pathways are mediated by autophagy-related proteins called ATGs and their associated enzymes. There are three commonly described forms of autophagy: macroautophagy, microautophagy and chaperone-mediated autophagy (CMA). Macroautophagy is the major pathway and primarily eradicates damaged or malformed organelles or proteins. In general, it involves the detachment of a portion of the endoplasmic reticulum (ER) called the phagophore, which elongates to form a double membrane organelle known as an autophagosome, which captures the cellular components to be degraded. The expansion of the autophagosomal membranes involves the incorporation of cytosolic microtubule-associated protein 1 - light chain 3 (MAP1LC3 - hereafter called LC3 only) in the membrane of the growing autophagosome. LC3, the mammalian homolog of the yeast ATG8 gene, is diffused throughout the cytoplasm (LC3 forms LC3-I). LC3-I assumes the LC3-II form when it is added to phosphatidylethanolamine, which is incorporated into the autophagosome. The autophagosome moves along microtubules and fuses to a lysosome to form an autolysosome or autophagolysosome, where the cellular content is degraded with acidic lysosomal hydrolases (Jung et al., 2010). Lysosomal permeases return the products of digestion into the cytosol, such as amino acids, lipids, nucleosides and carbohydrates, where they will be available for structural and metabolic pathways (Rabinowitz and White, 2010).

## Autophagy and its relationship with mTOR and TSC

Rapamycin treatment or induced starvation in human cells and mouse embryonic cells (MEFs) results in the dephosphorylation of ULK1, restoring autophagy (Hung et al., 2012). Furthermore, glucose starvation reduces ATP levels and activates AMPK, which is potentiated by LKB1, a protein kinase that phosphorylates AMPK. Activated AMPK inhibits mTORC1 and, as a consequence, positively regulates autophagy, thus making this pathway critical for monitoring cellular energy status and stress conditions. Moreover, autophagy can be induced by suppressing growth factor signaling pathways. The growth factor signaling in the IGF-1-PI3K-Akt pathway regulates mTORC1 to negatively regulate autophagy (Jung et al., 2010).

Chaperone-mediated autophagy (CMA) selectively degrades cytosolic proteins in lysosomes and contributes to the maintenance of proteostasis and cellular adaptation to stress. CMA substrates are delivered by a cytosolic chaperone to the surface of the lysosome, where they are unfolded and internalized through a membrane translocation complex. In murine models, lysosomal mTORC2 and Akt regulate the activity of CMA (Arias et al., 2015). Recently, the cochaperone BAG3 has been shown to coordinate protein synthesis and autophagy through the spatial regulation of mTORC1. BAG3 acts on the recruitment of the TSC complexes that inhibit the positive regulation of mTORC1 in the synthesis of cytoskeleton-associated actin fibers. In addition, when protein synthesis is necessary, BAG3 mediates the sequestration of the TSC complex, alleviating the inhibition of the mTORC1 that remains in the cytoplasm. In human muscle, an association of TSC1 with the exercise-induced cytoskeleton was described, indicating the coincidental activation of mTORC1 in the cytoplasm (Kathage et al., 2017).

Given that mTORC1 is a key inhibitor of autophagy through direct phosphorylation of ULK1, studying the states of TSC deficiency may provide an opportunity to investigate the implications of autophagy dysregulation in human pathophysiology (Kim et al., 2011). Through genetic and pharmacological inhibition of autophagy, it was possible to verify that TSC2-deficient tumor cells derived from LAM could be dependent on autophagy to survive. The induction of autophagy by mTOR inhibitors may enhance hamartoma cell adaptations to stress through a type of dormancy, in which proliferation is blocked due to inhibited mTORC1-mediated protein translation, leading to survival over time due to the activation of autophagy (Yu et al., 2010). This mechanism is important when considering the role of autophagy in cancer. Defects in autophagy may contribute to cell transformation and the initiation of tumorigenesis by causing the accumulation of defective proteins and organelles, thereby increasing oxidative stress and genetic instability. Parkhito *et al.* showed that lowering autophagy blocked TSC tumorigenesis across genetic down-regulation of p62/sequestosome 1 (SQSTM1), the autophagy substrate that accumulates in TSC tumors as a consequence of low autophagy levels. This substrate strongly inhibited the growth of TSC2-null xenografted tumors, demonstrating that autophagy is a critical component of TSC tumorigenesis and suggesting that mTORC1 inhibitors may have autophagy-dependent prosurvival effects in TSC, and revealing two distinct therapeutic targets for TSC: autophagy and the autophagy target p62/SQSTM1. (Parkhitko et al., 2011). More recently, p62 has also been associated with the maintenance of intracellular pools of glutamine, glutamate and glutathione, necessary to limit mitochondrial dysfunction in tumor cells with hyper activated mTORC1, being suggested as a possible therapeutic target in these tumors (Lam et al., 2017).

In summary, there is a consensus that autophagy acts as a chemopreventive mechanism in normal cells to prevent their transformation. In contrast to cells in normal tissues, the tumor and surrounding environment are often chronically deprived of nutrients, growth factors, and oxygen as a result of abnormal vascularization. In this context, autophagy may support tumor growth and the adaptation of tumor cells to metabolic stress as a mechanism to provide nutritional support (Rabinowitz and White, 2010). Thus, depending on the disease status, autophagy can either be beneficial or detrimental. For instance, the activation of the ULK1 pathway by compounds that act in the AMPK pathway can counteract inhibition of mTORC1 in *Tsc2*-knockdown neurons in mice suggesting that autophagy inducers can have therapeutic potential to treat TSC-associated neuronal pathologies (Di Nardo et al., 2014).

## Autophagy in TSC and other conditions with mTOR deficiency

To better understand the role of autophagy in TSC, we searched the PubMed database on May 16, 2019, to retrieve original articles describing the role of autophagy in TSC that were published in English and available in the literature between 2008 and 2019. The following keywords were used: “autophagy” and “tuberous sclerosis complex”, which resulted in the initial retrieval of 183 articles. We excluded the reviews, studies before 2008 and studies that did not relate to the scope of interest. Twenty-six articles were identified as studies related to the subject of this review and the studies are summarized in Table 1.

Autophagy seemed to play a role in various disease processes observed in TSC. For instance, insufficient autophagy in human melanocytes has been identified as responsible for hypopigmentation in specific sites in the skin, contributing to perhexiline the development of HM, which is one of the main clinical manifestations of TSC (Yang et al., 2018). Furthermore, some neuronal characteristics of TSC have been described as associated with autophagy. The level of autophagic proteins was different in dysmorphic neurons than it was in balloon cells (the term used for the abnormal cells observed in cortical tubers) or normal neurons, reflecting different degrees of activation of mTOR pathways in TSC cells (Miyahara et al., 2013). Overactived mTOR, together with impaired autophagy, may produce an excess of synaptic protein in neurons of patients with autism spectrum disorders (ASD, a common morbidity in TSC patients). This finding suggests that these two mechanisms may underlie the synaptic pathology of ASD (Tang et al., 2014). Injured autophagy may also contribute to epileptogenesis. The suppression of autophagy in the brain tissues of conditional *TSC1*-knockout mice resulted in aberrant mTOR activation and seizures. The conditional deletion of *Atg7* in mouse neurons was sufficient to promote the development of spontaneous seizures (McMahon et al., 2012).

The decrease of autophagy in some *in vitro* and *in vivo* studies was explained by the inactivation of *TSC1* or *TSC2* due to loss-of-function mutations, making the formation of the regulatory complex of mTORC1 infeasible. Due to this impaired regulation, the mTOR signaling pathway remained continuously active, leading to the uncontrolled proliferation of mutated cells and, as a consequence, to tumor formation. Interestingly, restoration of autophagy did not always affect tumors. In some cases, autophagy-related mTOR overactivation led to endoplasmatic reticulum (ER) stress and unfolded protein response (UPR) activation and to the formation of intracellular protein aggregates. These aggregated proteins contributed to cellular toxicity in different TSC cell culture models (Babcock et al., 2013; Di Nardo et al., 2009). Additionally, autophagy induction promoted tumor cell survival by enabling continuous cell growth in the tumoral microenvironment, which is generally characterized by starvation.

A few studies have also assessed the effect of dietary, non-pharmacological approaches associated with autophagy induction in TSC-associated tumors. The pro-autophagic metabolic interventions studied include glucose deprivation, ketogenic diet (KD) and carbohydrate restrictions. Harputlugil et al. (2014) showed that TSC1 was fundamental to the hepatoprotective effect played by protein restriction in ischemia-reperfusion injury. In agreement with this, TSC2-deficient MEFs were hypersensitive to amino acids starvation and hypoxia, in an ATG7-dependent manner. Indeed, the knockdown of *Atg7* in *Tsc2* ^+/+^ cells sensitized them to amino acid deprivation (Ng et al., 2011). Considering the link between TSC and neoplasias, in a study with 5 TSC patients, ketogenic diet did not produce beneficial effects to patients with TSC-related tumors, with no signs of growth suppression or tumor regression (Chu-Shore and Thiele, 2010). Considering the management of epilepsy in children with TSC, cognition and behavior were improved after ketogenic diet initiation, in addition to reducing seizure frequency (Park *et al.*, 2017). However, it is important to mention that autophagy was not measured in these models using ketogenic diet.

The increase in the rate of glycolysis in parallel with the reduction of oxidative phosphorylation is a typical hallmark of tumorigenesis (DeBerardinis et al., 2008). The overactivation of PI3k/Akt/mTOR pathway promotes glycolysis and glucose-dependence, leading *TSC1/TSC2* mutant tumor cells entering apoptosis after glucose withdraw (Maher et al., 2004). Indeed, *TSC2* suppresses apoptosis in contexts of energy deprivation (Inoki et al., 2003). Using a TSC tumor xenograft model Jiang *et al.* showed that animals receiving 2-deoxyglucose (2-DG, a modified form of glucose that cannot be used for glycolysis) showed reduced proliferation of tumor cells and the smallest tumors comparing to animals receiving Western-style diet or unrestricted carbohydrate-free. In addition, tumors from animals exposed to carb-free diets were larger and showed areas of necrosis and inflammation. Alternative energy substrates such as ketone bodies and monounsaturated oleic acid supported the growth of *Tsc2* ^*-/-*^ cells *in vitro*. This result suggests that glycolytic inhibition and glucose deprivation may be considered in TSC therapy (Jiang X *et al.*, 2011). However, it is important to keep in mind that pro-autophagic interventions used in these studies and other strategies to induce autophagy may modulate not only this mechanism, but also others including basal metabolism. Thus, attributing the effect of these interventions exclusively and directly to autophagy can lead to a misunderstanding if appropriated methodologies are not used. In fact, many studies with an ‘autophagy-centric’ bias do not consider other mechanisms. This aspect has to be lead into consideration in studies that aim to assess the effect of pro-autophagy strategies to treat TSC tumors.

## TSC, UPR and autophagy

High basal levels of ER stress were detected in different TSC-deficient cells (MEF cells, oligodendrocyte lineage and ELT3 leiomyoma rat smooth muscle cell line) (Ozcan et al., 2008; Babcock et al., 2013; Jiang et al., 2016). ER stress in mutant TSC cells is caused by increased cell proliferation which results in higher protein synthesis that overloads the ER capacity of protein folding. Moreover, autophagy impairment caused by mTOR overactivation in TSC-deficient cells also contributes to ER stress, since misfolded protein aggregates instead of being degraded by autophagy accumulates within ER lumen. In these cells, ER stress was detected due to activation of targets related to the UPR. The UPR is a cellular response triggered to relieve ER stress and reestablish protein homeostasis (Hetz and Papa, 2018). Therefore, UPR induces chaperones expression to improve ER capacity of protein folding, and the ERAD system (endoplasmic reticulum-associated degradation) to eliminate unfolded or misfolded proteins by proteasome or to eliminate protein aggregates by autophagy (Hwang and Qi, 2018).

Higher levels of UPR targets as PERK, XBP1, CHOP and GRP78 were detected in *TsC1* ^*-/-*^ MEFs when compared to wild-type cells, as well as increased UPR parameters in *TSC2*-mutant tumors (Ozcan et al., 2008; Qin et al., 2010; Tenkerian et al., 2015, Johnson et al., 2015). It was also observed that mTOR overactivaction of TSC-deficient cells results in c-MYC activation, which in turn, is able to induce UPR and ATF4 activation, by direct binding to its promoter, this may be a regulatory pathway (Babcock et al., 2013). TSC-deficient cells while presenting overactivation of mTORC1 show lower levels of mTORC2, as a compensatory negative feedback mechanism. It was shown that disruption of mTORC2 also contributed to the activation of the PERK - eIF2a arm of UPR, independently of mTORC1 (Tenkerian *et al.*, 2015).

Increased basal ER stress rendered TSC-deficient cells highly sensitive to cell death induced by pharmacological ER stressors as thapsigargin, tunicamycin and bortezomib (Ozcan et al., 2008, Johnson et al., 2015; Tenkerian et al., 2015). Increased apoptosis in TSC mutant versus wild-type cells suggests that these drugs can be a promising therapy option for disease control, since it would selectively kill tumor cells, while non-tumor and non-stressed cells would be able to cope to transient stress. Adjunct treatment with rapamycin was able to reduce UPR activation and rescued TSC mutant MEF cells from apoptosis induced by thapsigargin and tunicamycin (Ozcan *et al.*, 2008), as well rescued leiomyoma cells from apoptosis induced by bortezomib treatment (Babcock et al. 2013). These results confirm that overactivation of mTORC1 in TSC mutant cells is at least partly responsible for ER stress and UPR activation.

Since autophagy is triggered upon ER stress to eliminate excess proteins aggregates, and to mediate cell death under intense and prolongated stress, it is important to verify its relationship with ER stress on TSC-deficient cells. However, most of the studies that induced or checked ER stress in TSC deficient cells have not described autophagy levels. In TSC normal cells, drug-induced ER stress triggers autophagy-mediated cell death in MEF cells and ER stress-induced autophagy was attributed to the downregulation of mTOR pathway. On the other hand, when compared to wild-type cells, TSC -mutant cells with constitutive activation of mTOR were more resistance to ER stress-induced autophagy (Qin et al., 2010; Kang, et al., 2011).

Nelfinavir is an ER stressor drug which was tested on *Tsc2* ^*-/-*^ MEF cells and it was able to reduce mTOR signaling while increased autophagy levels, observed by decreased SQSTM1 protein and increased LC3 lipidation to the lower resolving LC3-II isoform. Chloroquine, an autophagy inhibitor, also enhanced nelfinavir-induced cell death in these cells. A combination of nelfinavir and chloroquine potentiated ER stress and affected autophagy resulting in *Tsc2* ^*-/-*^ cells death, while cells with normal expression of mTOR were tolerant. The combination of an ER-stressor drug and an autophagy inhibitor also seems to be a promising therapeutic option for TSC (Johnson et al., 2015).

## Autophagy therapies in TSC- and mTOR-deficient contexts

Many of the pathways that regulate autophagy are dysregulated in cancer development, and some therapeutic compounds have been designed to restore or inactivate these pathways (Table 1). Some of these compounds directly inhibit mTORC1, while others inhibit mTORC1 indirectly by reducing the nutritional support for cells or inhibiting the upstream targets in the mTOR signaling pathways (Yu et al., 2011). Some of these therapies leverage the combined therapeutic effect of the mTORC1 and mTORC2 complexes. However, therapies that focus on both complexes can induce considerable toxicity and drug resistance mechanisms. Recently, several studies have focused on the discovery of new compounds that act in these pathways, aiming to control exacerbated cell proliferation and autophagy. Among the alternative compounds that regulate autophagy by TSC2 are olaquindox, which induces autophagy and promotes apoptosis in HEK293 cells (Li et al., 2017). Perhexiline, niclosamide, amiodarone (approved drugs) and rottlerin (pharmacological reagent) inhibit mTORC1 signaling and stimulate autophagy (Balgi et al., 2009).

**Table:** 

TSC Sample	Goal of the manuscript	Autophagy findings	Reference
^A^MEF *TsC2* knock-out and knock-in	Coumponds perhexiline, niclosamide, amiodarone and rottlerin reversibly inhibit mTORC1 and stimulate autophagy	The chemicals activate autophagy in cells growing in nutrient rich conditions	Balgi et al., 2009
^A^MEF *TsC2* knock-out and knock-in, 621-101 and ^B^ELT3 *TsC2*-deficient cells, and renal lesions in mice heterozygous to *TsC2*	Autophagy is a critical component of TSC tumorigenesis, and the authors suggesting that rapamycin may have autophagy-dependent prosurvival effects	The combination of mTORC1 and autophagy inhibition was more effective than these isolated treatments, both for suppression of spontaneous tumor cells growth and of xenografts.	Parkhitko et al., 2011
Cortical tubers removed from 7 patients and 5 controls of cortical tissue taken from non‐TSC patients with epilepsy	Evaluation of induction of autophagy via mTOR that occurred in TSC-associated cortical tuber samples	Suppression of autophagy in cortical tubers presumably via the mTOR pathway	Miyahara *et al*., 2013
Brain tissues of conditional *TsC1* and phosphatase and tensin homolog knock-out mice	Autophagy was suppressed in samples investigated, which display seizures and aberrant mTOR activation; the conditional deletion of *Atg7* in mouse neurons is sufficient to promote of spontaneous seizures	The impaired autophagy contributes to epileptogenesis	McMahon *et al*., 2012
Primary cells from tuber samples of patients with TSC and frozen cells from a case of ^C^FCD	Defects in autophagy in ^C^FCD and in TSC share the altered mTOR pathway; this could be, in part, reversed *in vitro* by rapamycin	Abnormal activation of mTOR may contribute directly to a defect in autophagy in ^C^FCD and TSC	Yasin *et al*., 2013
^D^FAO cells, MCF-7 cells expressing constitutively active AKT (myr-AKT) and peroxisome-deficient human Zellweger cells	TSC has a role in the response to ^E^ROS in peroxisome and recognizes peroxisome as a signaling organelle involved in the regulation of mTORC1	TSC localized in peroxisomes functioned as a Rheb GTPase activator protein to suppress mTORC1 and induce autophagy	Zhang *et al*., 2013
Rat hippocampal neuronal cultures, ^A^MEFs, ^M^HEK293T cells, human TSC neurons collected from patients with intractable epilepsy	*Tsc2*-deficient neurons have increased autolysosome accumulation and autophagic flux despite mTORC1-dependent inhibition of ULK1; investigation of previously uncharacterized cellular mechanism that contributes to altered neuronal homeostasis in TSC disease	Loss of *Tsc2* gene in rat neurons results in autophagic activity via AMPK-dependent activation of ULK1; in *Tsc2*-knockdown neurons the AMPK activation is the dominant regulator of autophagy	Di Nardo *et al*., 2014
Transgenic mice with deletion of the *TsC1* gene by *CreLoxP,* breast tumor cells, isolated from mammary tumors created with the injection of these cells into nude mice	Creation of system that allows deletion of *TsC1* in tumor cells in an inducible manner; demonstration directly that deletion of *TsC1* and consequent activation of mTORC1 promoted tumor growth and metastasis, besides increased glucose starvation-induced autophagy and Akt activation	Glucose starvation-induced autophagy was increased significantly in *Tsc1*-null tumor cells, which could promote tumor cell survival and contribute to the increased tumor growth in vivo	Chen *et al*., 2014
MEFs *TsC2* knock-out and knock-in*, Tsc2*-null cystadenoma cell line, cells derived from mouse renal tumor	*TsC2*-null cells have distinctive autophagy-dependent ^F^PPP alterations, enhanced glucose uptake and utilization, decreased mitochondrial oxygen consumption, and increased mitochondrial ^E^ROS production; *TsC2*-deficient cells can be therapeutically targeted focusing on their metabolic vulnerabilities	^F^PPP is a key autophagy-dependent compensatory metabolic mechanism; ^F^PPP inhibition with ^G^6-AN in combination with autophagy inhibition suppressed proliferation and prompted the activation of ^H^NF-κB and ^I^CASP1 pathways in *TsC2*-deficient only	Parkhitko *et al*., 2014
Cell line stably expressing *TSC2* and ^J^LAM *TSC2* knock-out cells were obtained from patient sample, wild and knockout for *TsC2* ^A^MEFs	Rapamycin and resveratrol combination treatment blocked rapamycin-induced upregulation of autophagy and restored inhibition of Akt; this combination selectively promoted apoptosis of *TSC2*-deficient cells	Resveratrol caused inhibition of autophagy and targeting for apoptosis in *TSC2*-null cells	Alayev *et al*., 2015b
Dendritic spine and frozen samples from ^L^ASD patients and controls, postmortem tissue of the temporal lobe from patients with ^L^ASD, *TsC2* heterozygous mice; *TsC1* conditional knockout mouse line	mTOR-regulated autophagy is required for developmental spine pruning; and activation of neuronal autophagy corrects synaptic pathology and social behavior deficits in ^L^ASD models with hyperactivated mTOR	The activation of neuronal autophagy corrects synaptic pathology and social behavior deficits in ^L^ASD models with hyperactivated mTOR	Tang *et al*., 2014
Transgenic knock-in and knock-out mice for the *TsC1* and *YaP* genes by CreLoxP, cell line HEK293, MEFs knock-in and knock-out for *Tsc2.*	Regulation of ^N^YAP by mTOR and the autophagy pathway like a novel mechanism of growth control; this molecular mechanism was required for the tumorigenesis of TSC-related kidney lesions and in ^O^PEComas and the ^N^YAP may serve as a potential therapeutic target for TSC and other diseases with dysregulated mTOR activity	The data favor a model in which the control of ^N^YAP lysosomal degradation by mTOR matches ^N^YAP activity with nutrient availability in growth permissive conditions	Liang *et al*., 2014
^A^MEFs *TsC1* and *TsC2* knock-out and knock-in, ^B^ELT3 cells, Angiomyolipoma-derived tuberin-deficient cells; HeLa cells, GFP-LC3-expressing WI38 fibroblasts.	Hamartin interacts with ^P^PLK1; ^P^PLK1 protein levels are increased in hamartin and tuberin-deficient cells and ^J^LAM patient-derived specimens and that this increase is rapamycin-sensitive	PLK1 inhibition attenuated autophagy, and repressed the expression and protein levels of key autophagy genes and proteins and the protein levels of Bcl2 family members, suggesting that PLK1 regulates both autophagic and apoptotic responses	Valianou *et al*., 2015
CB17-SCID mice, ^B^ELT3 cells. For xenograft tumor establishment, 2.53106 cells were inoculated bilaterally into the posterior back region of mice	Combination of rapamycin and resveratrol is effective in reducing of tumors in TSC2-deficient in vivo; support of the model whereby the synergistic interaction of rapamycin and resveratrol results in a reduction of xenograft tumors	Rapamycin and resveratrol combination therapy not only arrested tumor growth but also by eliminating lesions, possibly through induction of apoptosis, besides also induced to suppression of autophagy induction	Alayev *et al*., 2015b
^A^MEF *TsC2* knock-out	The results indicate that an AMPK/p27 axis is promoting a survival mechanism that could explain in part the relapse of TSC tumors treated with rapamycin	Rapamycin induced increase of autophagic levels after 24h of serum deprivation; inhibition of AMPK with compound C inhibited basal levels of autophagy	Campos *et al*., 2016
Cardiac-specific *TsC2*-knock-out mice	Effects of hyperactivation of mTORC1 on cardiac function and structure; analysis of hearts revealed misalignment, aggregation and a decrease in the size and an increase in the number of mitochondria, but the mitochondrial function was maintained.	Autophagic flux was inhibited, while the phosphorylation level of ^S^S6 or ^R^4E-BP1 was increased; autophagy plays an important role in the maintenance of cardiac function and mitochondrial quantity and size in the heart	Taneike *et al*., 2016
MIN6 cell line; ^A^MEF knock-out *TsC1* and *TsC2,* and ^M^HEK293T cells.	Role of lysine acetylation in *TSC2,* in the regulation of mTORC1, autophagy and cell proliferation; effects of treatments with nicotinamide and resveratrol on mTORC1 signaling and autophagy modulation are *TSC2*-dependent	Nicotinamide increased TSC2 acetylation, and lead to mTORC1 activation and cell proliferation. In contrast, resveratrol avoided *TSC2* acetylation, inhibiting mTORC1 signaling and promoting autophagy	García-Aguilar *et al*., 2016
A7r5 rat skeletal muscle biopsies from musculus vastus lateralis collected 72 h after the last training activity, and post-exercise biopsies collected 45 min after acute resistance exercise	Cochaperone BAG3 stimulates translation through spatial regulation of mTORC1, inhibiting and recruiting the TSC complex to the cytoskeleton, where autophagy is initiated; mTORC1 inhibition in the remaining cytoplasm is relieved and translation efficiency increased	BAG3 insufficiency results in a severe imbalance of protein synthesis and protein degradation, and in autophagic levels	Kathage *et al*., 2017
^M^HEK293 cells	*TSC2* acted as a negative regulator of autophagy after olaquindox treatment, and also played a pro-apoptotic function	Olaquindox induced autophagy by reducing *TSC2* expression in ^M^HEK293 cells	Li *et al*., 2017
HeLa cells, primary human fibroblasts, human diploid fibroblasts, ^A^MEF knock-out *TsC2* and wild-type	During the acquisition of senescence occurs the constitutive activation of mTORC1, which is resistant to both serum and amino acid starvation; persistent mTORC1 simultaneously prevents senescent cells from realizing their full autophagic potential, which would otherwise lead to cell death	Constitutive mTOR activity in senescent cells was supported by high levels of autophagy, and increased autophagy contributes to mTORC1 deregulation and to the survival of senescent cells during starvation	Carroll *et al*., 2017
The generation of a *TSC*-cell model by isolating ^U^NSPCs from the brain of six-week-old *TsC1* mice.	Migration deficit observed in *Tsc1*-deficient ^U^NSPCs depends on the state of TFEB activation; treatments that promote ^V^TEFB nuclear translocation restore *Tsc1*-deficient ^U^NSPCs migation independently of mTORC1	^V^TFEB overexpression has been shown to reactivate autophagy and restore radial migration in new-born neurons where those processes were impaired	Magini A *et al*., 2017
^A^MEFs *TsC2* knock-out and knock-in and controls	General autophagy induction after uncoupling of oxidative phosphorylation by ^X^CCCP agent, and your importance in PINK1/parkin regulation which allows the directioning of uncounpled mitochondria to autophagahy degradation	Stimulation by ^X^CCCP resulted in increased of ^Y^LC3B-II protein in controls cells when compared to *TsC2*-deficient cells; *TsC2*-deficient cells showed less autophagy	Bartolomé A *et al*., 2017
Paraffin-embedded sections from skin lesions of ^W^HM from TSC patients and samples from corresponding sites of healthy donors; normal ^Z^HEMn-MP from moderately pigmented donors	Insufficient autophagy leads to reduced pigmentation in *TSC2*- silenced melanocytes; dysregulated autophagy contributes to hypopigmentation in patients with TSC in response to mTOR hyperactivation; enhancing both mTOR-dependent and -independent autophagy stands to improve depigmentation in TSC-model melanocytes	The results suggest that insufficient autophagy is a likely contributor to epidermal pigmentation abnormalities resulting in the hypomelanotic macules that are hallmarks of TSC.	Yang *et al*., 2018

^A^MEF: mouse embryonic fibroblasts; ^B^ELT3: Eker rat uterine leiomyoma-derived cells; ^C^FCD: focal cortical dysplasia; ^D^FAO: rat liver; ^E^ROS: reactive oxygen species; ^F^PPP: pentose phosphate pathway; ^G^6-AN: 6-aminonicotinamide; ^H^NF-κB: nuclear factor kappa B; ^I^CASP1: Caspase-1; ^J^LAM: Limfangioleiomyomatoses; ^L^ASD: autism spectrum disease; ^M^HEK293T: human embryonic kidney 293; ^N^YAP: hippo-Yes-associated protein 1 pathway; ^O^PEComas: tumours showing perivascular epithelioid cell differentiation; ^P^PLK1: polo-like kinase 1; ^Q^Bcl-2: B-cell lymphoma 2; ^R^4E-BP1: eukaryotic initiation factor 4E -binding protein 1; ^S^S6: ribosomal protein; ^T^MIN6: mouse insulinoma 6; ^U^NSPCs: neural stem/progenitor cells; ^V^TFEB: transcription factor EB; ^X^CCCP: carbonyl cyanide m-chlorophenyl hydrazone; ^Y^LC3: microtubule-associated protein 1A/1B-light chain 3; ^W^HM: hypopigmented macules; ^Z^HEMn-MP: human neonatal epidermal melanocytes.

In most cases, rapamycin treatment leads to the restoration of autophagy in tumor cells. This restoration can stop tumor growth in some cases. However, tumors may regrow after prolonged treatment with rapamycin due to the inhibition of the AMPK pathway, as demonstrated in MEFs from the Tsc2-null model (Campos et al., 2016). The impairment of autophagic flux and the accumulation of autophagosomes observed in the Tsc2-KD neurons of mice were also dependent on the AMPK pathway. After treatment with rapamycin, LC3-II accumulation and increased AMPK-ULK1 activation revealed that this accumulation of autolysosomes was insensitive to rapamycin, indicating a mTORC1-independent mechanism regulating autophagy (Di Nardo et al., 2014). Indeed, AMPK can directly activate the ULK1 complex in an mTOR-independent manner (Kim et al., 2011). Conversely, there is evidence suggesting that persistent mTORC1 signaling in the TSC context reduces the capacity of senescent cells to undergo autophagy, which leads to cell death. This seemingly contradictory role for autophagy as a prosurvival and cell death mechanism of senescent cells is a phenomenon that may also contribute to the tumorigenesis and neurodegeneration in TSC conditions (Carroll et al., 2017).

**Figure 2 -:**
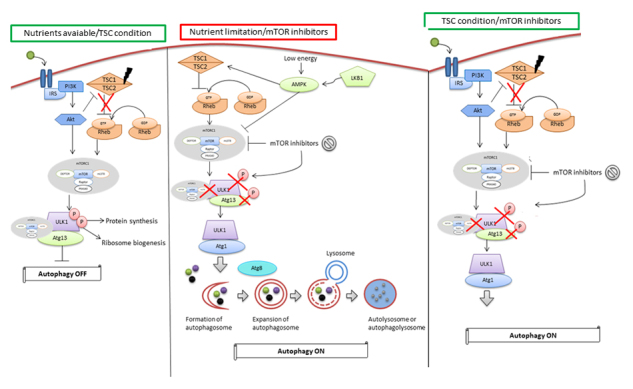
Autophagy signaling with nutrients available or TSC condition and with nutrient limitation or presence of mTOR inhibitors. In this first scenario, the TSC complex is unable to prevent to conversion of Rheb-GDP to Rheb-GTP causing the activation of mTORC1. mTORC1 activated causes inactivation of ULK1 complex through the phosphorylation of ULK1 and ATG13, thus suppressing autophagy and allowed the protein synthesis and ribosome biogenesis. Conversely, in the second scenario, under nutrient limitations or with the presence of mTOR inhibitors, mTORC1 is inactivated, which enables the ULK1 complex to be activated together with Atg proteins which triggers the formation of doble membrane vesicule called autophagosomes around the cell content that need to be degraded. Autophagosome merges with lysosome forming autolysome or autophagolysosome that degrades this vesicule internal cell content. In parallel to autophagy induction, the inactivation of mTORC inhibits cell growth.

Metformin and resveratrol have been given greater attention in recent years as possible means to stimulate autophagy in TSC. Metformin is an antihyperglycemic agent used for the treatment of noninsulin-dependent diabetes mellitus. The exact mechanism of action of metformin is not well elucidated, but a possible inhibitory effect on the mTOR signaling pathway has been recognized (Amin et al., 2018). Inhibition of mTORC1 by metformin can occur (a) through the phosphorylation and inhibition of Raptor and (b) through the activation of the *TSC1* and *TSC2* genes (Howell et al., 2017) (Figure 2). Normal and non-TSC2 (*TsC2* ^*-/-*^ ) embryonic mouse cells treated with metformin showed an upregulation of mTORC1 (Kalender et al., 2010). In the tumor model of mice with heterozygous mutations in *TSC2* (*Tsc2* ^*+/-*^ ) there was no reduction in tumor size after metformin treatment compared with those treated with rapamycin, suggesting limited therapeutic benefits of metformin in treating hamartomas (Auricchio et al., 2012). In murine kidney *TsC2* ^*+/-*^ tumors, metformin was able to reduce mTOR signaling only in normal tissues but not in tumor cells (Dowling et al., 2016). In liver cells of the same model, metformin inhibited mTOR signaling through a mechanism involving the AMPK pathway and the TSC complex (Howell *et al.*, 2017). None of these studies assessed autophagy after treatment. Some studies have raised the prospect of metformin use to suppress the initiation and recurrence of TSC-associated tumors, which would induce fewer side effects and have a lower cost than other mTOR inhibitors (Amin *et al.*, 2019).

Resveratrol is another compound investigated for its effect on mTOR inhibition and on the stimulation of autophagy and apoptosis. Resveratrol (3,5,4’-trihydroxystilbene) is a polyphenolic phytoalexin derived from stilbene. The compound is present in high concentrations in red wine, the imbibing of which has been associated with a lower incidence of heart disease. Other benefits of resveratrol include its anti-inflammatory, antioxidant (Wishart et al., 2018) and neuroprotective effects (Quincozes-Santos et al., 2013). It has been shown to regulate cell proliferation, apoptosis, and angiogenesis and to ameliorate DNA damage (Alayev et al., 2015), and regulate mitochondrial activity, which is important for the treatment of obesity and diabetes (Agarwal and Baur, 2011). Similar to metformin, resveratrol suppresses the activity of mTORC1 through the AMPK pathway (Wang et al., 2018), and induction of autophagy by resveratrol has been shown to occur directly via the mTOR-ULK1 pathway (Park et al., 2016). In addition, resveratrol inhibits the activation of many proteins upstream of the mTORC1/S6K1 signaling pathway, including PI3K and can modulate autophagy by directly inhibiting S6K1 activation (Armour et al., 2009) (see Figure 1). Additionally, resveratrol seems to also regulate the activation of mTORC2 (Gurusamy et al., 2010). Recent work has shown that resveratrol is capable of inducing apoptosis and autophagy in breast cancer tumor cells resistant to cisplatin treatment, non-small cell lung cancer cells and renal cell carcinoma cells (Park *et al.*, 2016; Chang et al., 2017; Liu et al., 2018; Wang *et al.*, 2018). Because of the ability of resveratrol to modulate the autophagy and apoptosis pathways, its efficacy was investigated in the context of TSC. It has been reported that resveratrol prevented the positive regulation of autophagy induced by rapamycin, inhibiting the cleavage of LC3 and the formation of autophagosomes in *TSC2* ^*-/-*^ cells. In this context, resveratrol was able to prevent p62 degradation in a *TSC2*-dependent manner (Alayev *et al.*, 2014). A combination of rapamycin and resveratrol tested in an animal model was able to specifically inhibit the PI3K/Akt/mTORC1 signaling pathway, activating apoptosis and reducing cell survival in a *TSC2* ^*-/-*^ xenograft tumor model of LAM but not in cells expressing *TSC2* (Alayev *et al.*, 2015).

## New treatment alternatives focused on autophagy modulation

A few treatment initiatives have already been reported for tumors with mTORC1 aberrant activation. These alternatives primarily target metabolic compensatory mechanisms triggered by autophagy inhibition but not solely through mTORC1 inhibition. The first example was published in the report by Parkhitko et al. (2014), who used chloroquine, a molecule that blocks lysosome-autophagosome fusion and lysosomal function, to suppress macroautophagy and chaperone-mediated autophagy. In this study, the authors demonstrate that TSC2-null cells have distinctive autophagy-dependent pentose phosphate pathway (PPP) alterations. With this in mind, they directly targeted mTORC1-independent autophagy by antimetabolite 6-aminonicotinamide. The authors showed a 40% reduction in cell proliferation after 96 h of treatment. In *Tsc* ^*+/-*^ mice with spontaneously formed cystoadenomas, the authors showed a 50% reduction in macrolesions and microlesions after 4 months of treatment (Parkhitko *et al.*, 2014). The second example was related to Polo-like kinase 1 (PLK1) inhibitors. These compounds were also shown to be potential therapeutic agents for the treatment of tumors with dysregulated mTORC1 signaling. The pharmacological inhibition of PLK1 by the small molecule BI-2536 significantly decreased the viability and clonogenic survival of TSC1- and TSC2-deficient cells in vitro. This inhibition of PLK1 was associated with increased apoptosis, suggesting that this kinase can regulate both autophagy and apoptosis (Valianou et al., 2015).

## Conclusions and perspectives

Tuberous Sclerosis Complex, although relatively rare, is the second most common disorder in the genodermatoses category of disorders. The disease can manifest in the early stages of life and often results in social exclusion and vulnerability at the medical, psychological and cultural levels. Severe epilepsy disorder may occur in very young patients. In addition, there is increased morbidity and a reduction in life expectancy, especially when hamartomas related to the syndrome emerge, especially as intracardiac and brain tumors. To date, mTOR inhibitors have been widely used to attenuate the clinical manifestations of the disease. However, several challenges still exist in the management of TSC using rapalogs, including their cost, side effects, definition of the ideal timing of treatment initiation for different disease symptoms and a better understanding of partial efficacy of these drugs in certain applications. In this scenario, alternative or combined treatments must be sought, and modulators of autophagy are promising candidates. The mTOR inhibitor-induced autophagy in tumor cells may have a dual effect by either interrupting or enhancing tumor growth. Currently, it is a consensus that autophagy is fundamental to control cell homeostasis and tissue functioning since its loss has been associated with the development of several pathologies. Autophagy deficiency has been observed in the early steps of tumor initiation, for instance. In the other hand, the adaptability to stress provided by autophagy can contribute to metabolic adaptation and tumor growth in advanced stages of carcinogenesis (Amaravadi et al., 2019). *TCS2*-deficient fibroblasts, for instance, are more dependent on exogenous nutrients, and the knockout of *ATG5* reduced even more cell adaptability to nutrient deprivation (Filippakis et al., 2018). In animal models, the above-mentioned dynamic of autophagy in cancer is reproducible, but the evidence from human samples suggests that this process is more complex (Galluzzi et al., 2015). Actually, it seems that the dominant effect played by autophagy (i.e. whether chemoprevention or tumor supporting) may depend on the tumor type, genetic and epigenetic background, the event involved in the carcinogenesis, among other variables. The fact is that the paradoxical role of autophagy in promoting or suppressing TSC tumorigenesis and modulating other clinical behaviors is inherent to the functioning of the mechanism, which controls multiple steps and, in the other hand, is controlled by several others (Amaravadi *et al.*, 2019). The large number of mechanisms controlling and being controlled by TSC complex and mTOR adds even more variables to the models (Filippakis *et al.*, 2018). These concepts must be kept in mind constantly in the discussion of pharmacological or non-pharmacological strategies that induce autophagy, either through the inhibition of mTOR or through other mechanisms. In several studies, however, a direct role has been attributed to autophagy even when the mechanism is not properly or exclusively assessed. This occurs, for instance, in studies showing that Rapamycin is effective in controlling tumor growth and/or reducing tumor volume in animal models (Kenerson et al., 2005; Lee et al., 2009) and in TSC patients (Cabrera-López et al., 2012; Mao et al., 2017; Li et al., 2019). Indeed, these evidences suggest that the chemopreventive role played by autophagy could be dominant. But other studies challenge the establishment of a unique conclusion, as exemplified by the observation that TSC individuals, despite not showing the altered incidence of renal tumors in relation to controls, developed renal cancer too much earlier in life (Peron et al., 2016). 

Therefore, two aspects need to be raised at this point. The first one concerns to the multitude of mechanisms controlled by TSC complex and mTOR in the interface between health and disease, from autophagy to protein synthesis, the activity of immune cells, cell metabolism, cell growth, and death (Boutouja et al. 2019). Attributing the effect of TSC mutations or Rapamycin exclusively to autophagy can be a misinterpretation. The second point is that tumors and other pathologies are not coordinated by a unique cell type or mechanism. Tumor microenvironment, for instance, is formed by dozens of cell types, including normal, immune and mesenchymal cells. The autophagy of all these players might be modulated when this mechanism is activated, and a growing body of recent evidence has shed some light on the crosstalk between tumor cells autophagy and the tumor microenvironment (Janji et al., 2018). Notwithstanding, the dominant role played by autophagy modulation, in this context, may depend not only on the genetic status of *TSC1/TSC2* but also the metabolism, the immune status, endocrine functions and other signaling molecules. With this in mind, it is important to maintain the criticism when interpreting results from mTOR inhibitors or metabolism modifications (Medvetz et al., 2015). In this sense, testing compounds that modulate autophagy in a more specific manner or animal models with the co-occurrence of *TSC1/TSC2* and *ATG* mutations could provide more accurate evidence regarding the role of autophagy in TSC (Filippakis et al., 2018). And, in addition to Rapamycin, new classes of mTOR inhibitors and compounds that modulate other abovementioned mechanisms could be combined to reach optimal clinical responses.

## Supplementary Material

The following online material is available for this article:

Table S1 - Clinical and Genetic criteria for the diagnosis of Tuberous Sclerosis Complex.

Table S2 - The main mTOR inhibitors tested in patients with TSC-related manifestations.

## References

[B1] Adriaensen ME, Schaefer-Prokop CM, Duyndam DA, Zonnenberg BA, Prokop M (2011). Radiological evidence of lymphangioleiomyomatosis in female and male patients with tuberous sclerosis complex. Clin Radiol.

[B2] Adriaensen ME, Schaefer-Prokop CM, Stijnen T, Duyndam DA, Zonnenberg BA, Prokop M (2009). Prevalence of subependymal giant cell tumors in patients with tuberous sclerosis and a review of the literature. Eur J Neurol.

[B3] Agarwal B, Baur JA (2011). Resveratrol and life extension. Ann N Y Acad Sci.

[B4] Alayev A, Berger SM, Holz MK (2015). Resveratrol as a novel treatment for diseases with mTOR pathway hyperactivation. Ann N Y Acad Sci.

[B5] Alayev A, Salamon RS, Sun Y, Schwartz NS, Li C, Yu JJ, Holz MK (2015). Effects of combining rapamycin and resveratrol on apoptosis and growth of TSC2-deficient xenograft tumors. Am J Respir Cell Mol Biol.

[B6] Alayev A, Sun Y, Snyder RB, Berger SM, Yu JJ, Holz MK (2014). Resveratrol prevents rapamycin-induced upregulation of autophagy and selectively induces apoptosis in TSC2-deficient cells. Cell Cycle.

[B7] Altmann J, Kiver V, Henrich W, Weichert A (2019). Clinical outcome of prenatally suspected cardiac rhabdomyomas of the fetus. J Perinat Med.

[B8] Amaravadi RK, Kimmelman AC, Debnath J (2019). Targeting Autophagy in Cancer: Recent Advances and Future Directions. Cancer Discov.

[B9] Amin S, Lux A, O’Callaghan F (2019). The journey of Metformin from glycaemic control to mTOR inhibition and the suppression of tumour growth. Br J Clin Pharmacol.

[B10] Amin S, Mallick AA, Edwards H, Lux A, Laugharne M, Likeman M, Khan A, O’Callaghan F (2018). A randomised, double-blind, parallel group, placebo-controlled trial of Metformin in Tuberous Sclerosis Complex. Arch Dis Child.

[B11] Arias E, Koga H, Diaz A, Mocholi E, Patel B, Cuervo AM (2015). Lysosomal mTORC2/PHLPP1/Akt regulate chaperone-mediated autophagy. Mol Cell.

[B12] Armour SM, Baur JA, Hsieh SN, Land-Bracha A, Thomas SM, Sinclair DA (2009). Inhibition of mammalian S6 kinase by resveratrol suppresses autophagy. Aging.

[B13] Auricchio N, Malinowska I, Shaw R, Manning BD, Kwiatkowski DJ (2012). Therapeutic trial of metformin and bortezomib in a mouse model of tuberous sclerosis complex (TSC). PLoS One.

[B14] Babcock JT, Nguyen HB, He Y, Hendricks JW, Wek RC, Quilliam LA (2013). Mammalian target of Rapamycin Complex 1 (mTORC1) enhances Bortezomib-induced death in Tuberous Sclerosis Complex (TSC)-null cells by a c-MYC-dependent induction of the Unfolded Protein Response. J Biol Chem.

[B15] Balgi AD, Fonseca BD, Donohue E, Tsang TC, Lajoie P, Proud CG, Nabi IR, Roberge M (2009). Screen for chemical modulators of autophagy reveals novel therapeutic inhibitors of mTORC1 signaling. PLoS One.

[B16] Bartolomé A, García-Aguilar A, Asahara SI, Kido Y, Guillén C, Pjavani UB, Benito M (2017). MTORC1 regulates both general autophagy and mitophagy induction after oxidative phosphorylation uncoupling. Mol Cell Biol.

[B17] Bissler JJ, Christopher Kingswood JC, Radzikowska E, Zonnenberg BA, Frost M, Belousova E, Sauter M, Nonomura N, Brakemeier S, de Vries PJ (2013). Everolimus for angiomyolipoma associated with tuberous sclerosis complex or sporadic lymphangioleiomyomatosis (EXIST-2): a multicenter, randomized, double-blind, placebo-controlled trial. Lancet.

[B18] Bissler JJ, Franz DN, Frost MD, Belousova E, Bebin EM, Sparagana S, Berkowitz N, Ridolfi A, Kingswood JC (2018). The effect of everolimus on renal angiomyolipoma in pediatric patients with tuberous sclerosis being treated for subependymal giant cell astrocytoma. Pediatr Nephrol.

[B19] Bissler JJ, Kingswood JC, Radzikowska E, Zonnenberg BA, Belousova E, Frost MD, Sauter M, Brakemeier S, de Vries PJ, Berkowitz N (2017). Everolimus long-term use in patients with tuberous sclerosis complex: Four-year update of the EXIST-2 study. PLoS One.

[B20] Boutouja F, Stiehm CM, Platta HW (2019). mTOR: A cellular regulator interface in health and disease. Cells.

[B21] Cabrera-López C, Martí T, Catalá V, Torres F, Mateu S, Ballarín J, Torra R (2012). Assessing the effectiveness of rapamycin on angiomyolipoma in tuberous sclerosis: a two-year trial. Orphanet J Rare Dis.

[B22] Campos T, Ziehe J, Fuentes-Villalobos F, Riquelme O, Peña D, Troncoso R, Lavandero S, Morin V, Pincheira R, Castro AF (2016). Rapamycin requires AMPK activity and p27 expression for promoting autophagy-dependent Tsc2-null cell survival. Biochim Biophys Acta.

[B23] Carroll B, Nelson G, Rabanal-Ruiz Y, Kucheryavenko O, Dunhill-Turner NA, Chesterman CC, Zahari O, Zhang T, Conduit SE, Mitchell CA (2017). Persistent mTORC1 signaling in cell senescence results from defects in amino acid and growth factor sensing. J Cell Biol.

[B24] Chang CH, Lee CY, Lu CC, Tsai FJ, Hsu YM, Tsao JW, Juan YN, Chiu HY, Yang JS, Wang CC (2017). Resveratrol-induced autophagy and apoptosis in cisplatin-resistant human oral cancer CAR cells: A key role of AMPK and Akt/mTOR signaling. Int J Oncol.

[B25] Chen Y, Wei H, Liu F, Guan JL (2014). Hyperactivation of mammalian target of rapamycin complex 1 (mTORC1) promotes breast cancer progression through enhancing glucose starvation-induced autophagy and Akt signaling. J Biol Chem.

[B26] Chu-Shore CJ, Thiele EA (2010). Tumor growth in patients with Tuberous Sclerosis Complex on the ketogenic diet. Brain Dev.

[B27] Cudzilo CJ, Szczesniak RD, Brody AS, Rattan MS, Krueger DA, Bissler JJ, Franz DN, McCormack FX, Young L (2013). Lymphangioleiomyomatosis screening in women with tuberous sclerosis. Chest.

[B28] DeBerardinis RJ, Lum JJ, Hatzivassiliou G, Thompson CB (2008). The biology of cancer: metabolic reprogramming fuels cell growth and proliferation. Cell Metabolism.

[B29] Dibble CC, Manning BD (2013). Signal integration by mTORC1 coordinates nutrient input with biosynthetic output. Nat Cell Biol.

[B30] Di Nardo A, Wertz MH, Kwiatkowski E, Tsai PT, Leech JD, Greene-Colozzi E, Goto J, Dilsiz P, Talos DM, Clish CB (2014). Neuronal Tsc1/2 complex controls autophagy through AMPK-dependent regulation of ULK1. Hum Mol Genet.

[B31] Di Nardo A, Kramvis I, Cho N, Sadowski A, Meikle L, Kwiatkowski DJ, Sahin M (2009). Tuberous sclerosis complex activity is required to control neuronal stress responses in an mTOR-dependent manner. J Neurosci.

[B32] Dowling RJ, Lam S, Bassi C, Mouaaz S, Aman A, Kiyota T, Al-Awar R, Goodwin PJ, Stambolic V (2016). Metformin pharmacokinetics in mouse tumors: Implications for human therapy. Cell Metab.

[B33] Filippakis H, Belaid A, Siroky B, Wu C, Alesi N, Hougard T, Nijmeh J, Lam HC, Henske EP (2018). Vps34-mediated macropinocytosis in Tuberous Sclerosis Complex 2-deficient cells supports tumorigenesis. Sci Rep.

[B34] Franz DN, Capal JK (2017). mTOR inhibitors in the pharmacologic management of tuberous sclerosis complex and their potential role in other rare neurodevelopmental disorders. Orphanet J Rare Dis.

[B35] Franz DN, Belousova E, Sparagana E, Bebin M, Frost MD, Kuperman R, Witt O, Kohrman MH, Flamini JR, Wu JY (2016). Long-term use of Everolimus in patients with Tuberous Sclerosis Complex: Final results from the EXIST-1 Study. PLoS One.

[B36] Franz DN, Leonard J, Tudor C, Chuck G, Care M, Sethuraman G, Dinopoulos A, Thomas G, Crone KR (2006). Rapamycin causes regression of astrocytomas in tuberous sclerosis complex. Ann Neurol.

[B37] Galluzzi L, Pietrocola F, Pedro JMB-S, Amaravadi RK, Baehrecke EH, Cecconi F, Codogno P, Debnath J, Gewirtz DA, Karantza V (2015). Autophagy in malignant transformation and cancer progression. EMBO J.

[B38] Gao X, Zhang Y, Arrazola P, Hino O, Kobayashi T, Yeung RS, Ru B, Pan D (2002). TSC tumour suppressor proteins antagonize amino-acid-TOR signaling. Nat Cell Biol.

[B39] García-Aguilar A, Guillén C, Nellist M, Bartolomé A, Benito M (2016). TSC2 N-terminal lysine acetylation status affects to its stability modulating mTORC1 signaling and autophagy. Biochim Biophys Acta.

[B40] Gurusamy N, Lekli I, Mukherjee S, Ray D, Ahsan MK, Gherghiceanu M, Popescu LM, Das DK (2010). Cardioprotection by resveratrol: a novel mechanism via autophagy involving the mTORC2 pathway. Cardiovasc Res.

[B41] Harputlugil E, Hine C, Vargas D, Robertson L, Manning BD, Mitchell JR (2014). The TSC complex is required for the benefits of dietary protein restriction on stress resistance in vivo. Cell Reports.

[B42] Hatano T, Egawa S (2020). Renal angiomyolipoma with tuberous sclerosis complex: How it differs from sporadic angiomyolipoma in both management and care. Asian J Surg.

[B43] Hay N, Sonenberg N (2004). Upstream and downstream of mTOR. Genes Dev.

[B44] Henske EP, Jóźwiak S, Kingswood JC, Sampson JR, Thiele EA (2016). Tuberous sclerosis complex. Nat Rev Dis Primers.

[B45] Hetz C, Papa FR (2018). The Unfolded Protein Response and cell fate control. Mol Cell.

[B46] Hosokawa N, Hara T, Kaizuka T, Kishi C, Takamura A, Miura Y, Iemura S, Natsume T, Takehana K, Yamada N (2009). Nutrient-dependent mTORC1 association with the ULK1-Atg13-FIP200 complex required for autophagy. Mol Biol Cell.

[B47] Howell JJ, Hellberg K, Turner M, Talbott G, Kolar MJ, Ross DS, Hoxhaj G, Saghatelian A, Shaw RJ, Manning BD (2017). Metformin inhibits hepatic mTORC1 signaling via dose-dependent mechanisms involving AMPK and the TSC Complex. Cell Metab.

[B48] Huang J, Manning BD (2008). The TSC1-TSC2 complex: a molecular switchboard controlling cell growth. Biochem J.

[B49] Huang J, Dibble CC, Matsuzaki M, Manning BD (2008). The TSC1-TSC2 complex is required for proper activation of mTOR complex 2. Mol Cell Biol.

[B50] Hung CM, Garcia-Haro L, Sparks CA, Guertin DA (2012). mTOR-dependent cell survival mechanisms. Cold Spring Harb Perspect Biol.

[B51] Hwang J, Qi L (2018). Quality Control in the Endoplasmic Reticulum: Crosstalk between ERAD and UPR pathways. Trends Biochem Sci.

[B52] Inoki K, Zhu T, Guan KL (2003). TSC2 mediates cellular energy response to control cell growth and survival. Cell.

[B53] Jacinto E, Loewith R, Schmidt A, Lin S, Rüegg MA, Hall A, Hall MN (2004). Mammalian TOR complex 2 controls the actin cytoskeleton and is rapamycin insensitive. Nat Cell Biol.

[B54] Janji B, Berchem G, Chouaib S (2018). Targeting autophagy in the tumor microenvironment: new challenges and opportunities for regulating tumor immunity. Front Immunol.

[B55] Jiang M, Liu L, He X, Wang H, Lin W, Wang H, Yoon SO, Wood TL, Lu RQ (2016). Regulation of PERK-eIF2a signalling by tuberous sclerosis complex-1 controls homoeostasis and survival of myelinating oligodendrocytes. Nat Commun.

[B56] Jiang X, Kenerson HL, Yeung RS (2011). Glucose deprivation in Tuberous Sclerosis Complex-related tumors. Cell Biosci.

[B57] Johnson CE, Hunt DK, Wiltshire M, Herbert TP, Sampson JR, Errington RJ, Davies DM, Tee AR (2015). Endoplasmic reticulum stress and cell death in mTORC1-overactive cells is induced by nelfinavir and enhanced by chloroquine. Mol Oncol.

[B58] Julien LA, Carriere A, Moreau J, Roux PP (2010). mTORC1-activated S6K1 phosphorylates Rictor on threonine 1135 and regulates mTORC2 signaling. Mol Cell Biol.

[B59] Jung CH, Ro SH, Cao J, Otto NM, Kim DH (2010). mTOR regulation of autophagy. FEBS Lett.

[B60] Kalender A, Selvaraj A, Kim SY, Gulati P, Brûlé S, Viollet B, Kemp BE, Bardeesy N, Dennis P, Schlager JJ (2010). Metformin, independent of AMPK, inhibits mTORC1 in a rag GTPase-dependent manner. Cell Metab.

[B61] Kang YJ, Lu MK, Guan KL (2011). The TSC1 and TSC2 tumor suppressors are required for proper ER stress response and protect cells from ER stress-induced apoptosis. Cell Death Diff.

[B62] Kathage B, Gehlert S, Ulbricht A, Lüdecke L, Tapia VE, Orfanos Z, Wenzel D, Bloch W, Volkmer R, Fleischmann BK (2017). The cochaperone BAG3 coordinates protein synthesis and autophagy under mechanical strain through spatial regulation of mTORC1. Biochim Biophys Acta Mol Cell Res.

[B63] Kenerson H, Dundon TA, Yeung RS (2005). Effects of rapamycin in the Eker rat model of Tuberous Sclerosis. Pediatr Res.

[B64] Kilincaslan A, Kok BE, Tekturk P, Yalcinkaya C, Ozkara C, Yapici Z (2017). Beneficial effects of Everolimus on Autism and Attention-Deficit/Hyperactivity Disorder Symptoms in a group of patients with Tuberous Sclerosis Complex. J Child Adolesc Psychopharmacol.

[B65] Kim J, Kundu M, Viollet B, Guan KL (2011). AMPK and mTOR regulate autophagy through direct phosphorylation of Ulk1. Nat Cell Biol.

[B66] Kingswood JC, Jozwiak S, Belousova ED, Frost MD, Kuperman RA, Bebin EM, Korf BR, Flamini JR, Kohrman MH, Sparagana SP (2014). The effect of everolimus on renal angiomyolipoma in patients with tuberous sclerosis complex being treated for subependymal giant cell astrocytoma: subgroup results from the randomized, placebo-controlled, Phase 3 trial EXIST-1. Nephrol Dial Transplant.

[B67] Koenig MK, Bell CS, Hebert AA, Roberson J, Samuels JA, Slopis JM, Tate P, Northrup H (2018). Efficacy and safety of topical rapamycin in patients with facial angiofibromas secondary to tuberous sclerosis complex: the treatment randomized clinical trial. JAMA Dermatol.

[B68] Lam CH, Baglini CV, Lope AL, Parkhitko AA, Liu HJ, Alesi N, Malinowska IA, Ebrahimi-Fakhari D, Saffari A, Yu JJ (2017). p62/SQSTM1cooperates with hyperactive mTORC1 to regulate glutathione production, maintain mitochondrial integrity and promote tumorigenesis. Cancer Res.

[B69] Lee N, Woodrum CL, Nobil AM, Rauktys AE, Messina MP, Dabora SL (2009). Rapamycin weekly maintenance dosing and the potential efficacy of combination Sorafenib plus Rapamycin but not Atorvastatin or Doxycycline in Tuberous Sclerosis preclinical models. BMC Pharmacol.

[B70] Li D, Zhao K, Yang X, Xiao X, Tang S (2017). TCS2 Increases Olaquindox-induced apoptosis by upregulation of ROS production and downregulation of autophagy in HEK293 cells. Molecules.

[B71] Li M, Zhou Y, Chen C, Yang T, Zhou S, Chen S, Wu Y, Cui Y (2019). Efficacy and safety of mTOR inhibitors (rapamycin and its analogues) for tuberous sclerosis complex: a meta-analysis. Orphanet J Rare Dis.

[B72] Liang N, Zhang C, Dill P, Panasyuk G, Pion D, Koka V, Gallazzini M, Olson EN, Lam H, Henske EP (2014). Regulation of YAP by mTOR and autophagy reveals a therapeutic target of tuberous sclerosis complex. J Exp Med.

[B73] Liu Q, Fang Q, Ji S, Han Z, Cheng W, Zhang H (2018). Resveratrol-mediated apoptosis in renal cell carcinoma via the p53/AMP-activated protein kinase/mammalian target of rapamycin autophagy signaling pathway. Mol Med Rep.

[B74] MacKeigan JP, Krueger DA (2015). Differentiating the mTOR inhibitors everolimus and sirolimus in the treatment of tuberous sclerosis complex. Neuro Oncol.

[B75] Magini A, Polchi A, Meo DD, Mariucci G, Sagini K, Marco FD, Cassano T, Giovagnoli S, Dolcetta D, Emiliani C (2017). TFEB activation restores migration ability to Tsc1-deficient adult neural stem/progenitor cells. Hum Mol Genet.

[B76] Maher JC, Krishan A, Lampidis TJ (2004). Greater cell cycle inhibition and cytotoxicity induced by 2-deoxy-D-glucose in tumor cells treated under hypoxic vs. aerobic conditions. Cancer Chemother Pharmacol.

[B77] Mao S, Long Q, Lin H, Liu J (2017). Rapamycin therapy for neonatal tuberous sclerosis complex with cardiac rhabdomyomas: A case report and review. Exp Ther Med.

[B78] McCormack FX, Inoue Y, Moss J, Singer LG, Strange C, Nakata K, Barker AF, Chapman JT, Brantly ML, Stocks JM (2011). Efficacy and safety of sirolimus in lymphangioleiomyomatosis. N Engl J Med.

[B79] McMahon J, Huang X, Yang J, Komatsu M, Yue Z, Qian J, Zhu X, Huang Y (2012). Impaired autophagy in neurons after disinhibition of mammalian target of rapamycin and its contribution to epileptogenesis. J Neurosci.

[B80] Medvetz D, Priolo C, Henske EP (2015). Therapeutic targeting of cellular metabolism incells with hyperactive mTORC1: A paradigm shift. Mol Cancer Res.

[B81] Menon S, Dibble CC, Talbott G, Hoxhaj G, Valvezan AJ, Takahashi H, Cantley LC, Manning BD (2014). Spatial control of the TSC complex integrates insulin and nutrient regulation of mTORC1 at the lysosome. Cell.

[B82] Miyahara H, Natsumeda M, Shiga A, Aoki H, Toyoshima Y, Zheng Y, Takeuchi R, Murakami H, Masuda H, Kameyama S (2013). Suppressed expression of autophagosomal protein LC3 in cortical tubers of tuberous sclerosis complex. Brain Pathol.

[B83] Nabbout R, Santos M, Rolland Y, Delalande O, Dulac O, Chiron C (1999). Early diagnosis of subependymal giant cell astrocytoma in children with tuberous sclerosis. J Neurol Neurosurg Psychiatry.

[B84] Ng S, Wu YT, Chen B, Zhou J, Shen HM (2011). Impaired Autophagy Due to Constitutive mTOR Activation Sensitizes TSC2-null Cells to Cell Death Under Stress. Autophagy.

[B85] Northrup H, Koenig MK, Pearson DA, Au KS, Adam MP, Ardinger HH, Pagon RA, Wallace SE (2020). Tuberous Sclerosis Complex. GeneReviews®.

[B86] Northrup H, Krueger DA (2013). Tuberous Sclerosis Complex diagnostic criteria update: Recommendations of the 2012 International Tuberous Sclerosis Complex Consensus Conference. Pediatr Neurol.

[B87] Ozcan U, Ozcan L, Yilmaz E, Düvel K, Sahin M, Manning BD, Hotamisligil GS (2008). Loss of the Tuberous Sclerosis Complex tumor suppressors triggers the Unfolded Protein Response to regulate insulin signaling and apoptosis. Mol Cell.

[B88] Park D, Jeong H, Lee MN, Koh A, Kwon O, Yang YR, Noh J, Suh PG, Park H, Ryu SH (2016). Resveratrol induces autophagy by directly inhibiting mTOR through ATP competition. Sci Rep.

[B89] Park S, Lee EJ, Eom S, Kang HC, Lee JS, Kim HD (2017). Ketogenic diet for the management of epilepsy associated with Tuberous Sclerosis Complex in children. J Epilepsy Res.

[B90] Parkhitko AA, Priolo C, Coloff JL, Yun J, Wu JJ, Mizumura K, Xu W, Malinowska IA, Yu J, Kwiatkowski DJ (2014). Autophagy-dependent metabolic reprogramming sensitizes TSC2-deficient cells to the antimetabolite 6-aminonicotinamide. Mol Cancer Res.

[B91] Parkhitko AA, Priolo C, Coloff JL, Yun J, Wu JJ, Mizumura K, Xu W, Malinowska IA, Yu J, Kwiatkowski DJ (2011). Tumorigenesis in tuberous sclerosis complex is autophagy and p62/sequestosome 1 (SQSTM1)-dependent. Proc Natl Acad Sci U S A.

[B92] Peron A, Vignoli A, La Briola F, Volpi A, Montanari E, Morenghi E, Ghelma F, Bulfamante G, Cefalo G, Canevini MP (2016). Do patients with Tuberous Sclerosis Complex have an increased risk for malignancies?. Am J Med Genet A.

[B93] Qin L, Wang Z, Tao L, Wang Y (2010). ER stress negatively regulates AKT/TSC/mTOR pathway to enhance autophagy. Autophagy.

[B94] Quincozes-Santos A, Bobermin LD, Latini A, Wajner M, Souza DO, Gonçalves CA, Gottfried C (2013). Resveratrol protects C6 astrocyte cell line against hydrogen peroxide-induced oxidative stress through heme oxygenase 1. PLoS One.

[B95] Rabinowitz JD, White E (2010). Autophagy and metabolism. Science.

[B96] Sahin M, Henske EP, Manning BD, Ess KC, Bissler JJ, Klann E, Kwiatkowski DJ, Roberds SL, Silva AJ, Hillaire-Clarke CS (2016). Advances and future directions for Tuberous Sclerosis Complex research: Recommendations from the 2015 Strategic Planning Conference. Pediatr Neurol.

[B97] Saucedo LJ, Gao X, Chiarelli DA, Li L, Pan D, Edgar BA (2003). RHEB promotes cell growth as a component of the insulin/TOR signalling network. Nat Cell Biol.

[B98] Shepherd CW, Scheithauer BW, Gomez MR, Altermatt HJ, Katzmann JA (1991). Subependymal giant cell astrocytoma: a clinical, pathological, and flow cytometric study. Neurosurgery.

[B99] Taneike M, Nishida K, Omiya S, Zarrinpashneh E, Misaka T, Kitazume-Taneike R, Austin R, Takaoka M, Yamaguchi O, Gambello MJ (2016). mTOR hyperactivation by ablation of Tuberous Sclerosis Complex 2 in the mouse heart induces cardiac dysfunction with the incfeased number of small mitochondria mediated through the down-regulation of autophagy. PLoS One.

[B100] Tang G, Gudsnuk K, Kuo SH, Cotrina ML, Rosoklija G, Sosunov A, Sonders MS, Kanter E, Castagna C, Yamamoto A (2014). Loss of mTOR-dependent macroautophagy causes autistic-like synaptic pruning deficits. Neuron.

[B101] Tenkerian C, Krishnamoorthy J, Mounir Z, Kazimierczak U, Khoutorsky A, Staschke KA, Kristof AS, Wang S, Hatzoglou M, Koromilas AE (2015). mTORC2 balances AKT activation and eIF2a Serine 51 phosphorylation to promote survival under stress. Mol Cancer Res.

[B102] Tjarks BJ, Gardner JM, Riddle ND (2019). Hamartomas of skin and soft tissue. Semin Diagn Pathol.

[B103] Trelinska J, Dachowska I, Kotulska K, Fendler W, Jozwiak S, Mlynarski W (2015). Complications of mammalian target of rapamycin inhibitor anticancer treatment among patients with tuberous sclerosis complex are common and occasionally life-threatening. Anticancer Drugs.

[B104] Tworetzky W, McElhinney DB, Margossian R, Moon-Grady AJ, Sallee D, Goldmuntz E, Van der Velde ME, Silverman NH, Allan LD (2003). Association between cardiac tumors and tuberous sclerosis in the fetus and neonate. Am J Cardiol.

[B105] Uysal SP, Sahin M (2020). Tuberous Sclerosis Complex: A review of the past, present and future. Turk J Med Sci.

[B106] Valianou M, Cox AM, Pichette B, Hartley S, Paladhi UR, Astrinidis A (2015). Pharmacological inhibition of Polo-like kinase 1 (PLK1) by BI-2536 decreases the viability and survival of hamartin and tuberin deficient cells via induction of apoptosis and attenuation of autophagy. Cell Cycle.

[B107] Xie J, Proud CG (2014). Signaling crosstalk between the mTOR complexes. Translation.

[B108] Xu KF, Xu W, Liu S, Yu J, Tian X, Yang Y, Wang ST, Zhang W, Feng R, Zhang T (2020). Lymphangioleiomyomatosis. Semin Respir Crit Care Med.

[B109] Yang F, Yang L, Wataya-Kaneda M, Hasegawa J, Yoshimori T, Tanemura A, Tsuruta D, Katayama I (2018). Dysregulation of autophagy in melanocytes contributes to hypopigmented macules in tuberous sclerosis complex. J Dermatol Sci.

[B110] Yasin AS, Ali AM, Tata M, Picker SR, Anderson GW, Latimer-Bowman E, Nicholson SL, Harkness W, Cross JH, Paine SML (2013). mTOR-dependent abnormalities in autophagy characterize human malformations of cortical development: evidence from focal cortical dysplasia and tuberous sclerosis. Acta Neuropathol.

[B111] Yu J, Parkhitko A, Henske EP (2011). Autophagy: an ‘Achilles’ heel of tumorigenesis in TSC and LAM. Autophagy.

[B112] Yu J, Parkhitko AA, Henske EP (2010). Mammalian target of rapamycin signaling and autophagy: roles in lymphangioleiomyomatosis therapy. Proc Am Thorac Soc.

[B113] Wang J, Li J, Cao N, Li Z, Han J, Li L (2018). Resveratrol, an activator of SIRT1, induces protective autophagy in non-small-cell lung cancer via inhibiting Akt/mTOR and activating p38-MAPK. Onco Targets Ther.

[B114] Wishart DS, Feunang YD, Guo AC, Lo EJ, Marcu A, Grant JR, Sajed T, Johnson D, Li C, Sayeeda Z (2018). DrugBank 5.0: a major update to the DrugBank database for 2018. Nucleic Acids Res.

[B115] Wullschleger S, Loewith R, Hall MN (2006). TOR signaling in growth and metabolism. Cell.

[B116] Zhang J, Kim J, Alexander A, Cai S, Tripathi DN, Dere R, Tee AR, Tait-Mulder J, Di Nardo A (2013). A tuberous sclerosis complex signalling node at the peroxisome regulates mTORC1 and autophagy in response to ROS. Nat Cell Biol.

[B117] Zou L, Liu Y, Pang L, Ju J, Shi Z, Zhang J, Chen X, Su X, Hu L, Shi X (2014). Efficacy and safety of rapamycin in treatment of children with epilepsy complicated with tuberous sclerosis. Zhonghua Er Ke Za Zhi.

