# Supplementary material for: The paradox of autophagy in Tuberous Sclerosis Complex
Source: Genet Mol Biol. 2021 Apr 5;44(2):e20200014. doi: 10.1590/1678-4685-GMB-2020-0014 (PMC8022228; doi:10.1590/1678-4685-GMB-2020-0014)
Supplement: Table S1 - [file 1415-4757-GMB-44-2-e20200014-s1.pdf]

Genetics and Molecular Biology
DOI: https://doi.org/ 10.1590/1678-4685-GMB-2020-0014

Supplementary material to: The paradox of autophagy in tuberous sclerosis complex

Table S1 - Clinical and Genetic criteria for the diagnosis of Tuberous Sclerosis Complex.

**Genetic diagnosis criteria**		
The identification of any pathogenic variant in the *TSC1* or *TSC2* genes in normal tissue DNA is an independent and sufficient criterion for diagnosis, independent of clinical findings. Pathogenic variants are defined as:		
(1) Variants that inactivate the function of TSC1 or TSC2 proteins;		
(2) Variants that clearly inactivate the function of the TSC1 or TSC1 proteins: variants that cause change in the reading phase or variants that cause premature stop codon;		
(3) Variants that prevent the synthesis of proteins: larges genomic deletions;		
(4) Silent variants whose effects on protein function can be determined by functional assay;		
**Clinical diagnosis criteria**		
Dermatological and dental features		
Key Features	Frequency	Onset symptoms
HM[A] ≥3, at least 5mm in diameter	Observed in about 90% of patients	At birth or childhood
Angiofibromas ≥3 or cephalic fibrous plaques	Angiofibromas occur in about 75% of patients; fibrous plaques in about 25%	Between 2 and 5 years old
Ungual fibromas ≥2	Frequency close to 20% in adults can reach 80%	Adolescence and adult life
Shagreen patches	Observed in about 50% of patients	First decade of life
Secondary features	Frequency	Onset symptoms
Confetti skin lesions, 1 to 3mm in diameter	Frequency ranges from 3% to 58% of patients	Varied
Cavities in dental enamel ≥3	Mlynarczyk G. reported in 100% of adult patients, in a total of 50	Adolescence and adult life
Intraoral fibromas ≥2	It occurs in about 20 to 50% of patients	Varied
Ophthalmic features		
Key Features	Frequency	Onset symptoms
Multiple retinal hamartomas	They are observed in 30 to 50%; it is not usual to find multiple lesions in the same patient	Childhood
Secondary features	Frequency	Onset symptoms
Achroman stain on the retina	Occurrence of 39% of patients	Varied

Central Nervous System features		
Key Features	Frequency	Onset symptoms
Cortical dysplasia	Cortical tubers observed in about 90% of patients	Fetal development
SEN and SEGA	SEN observed in 80% of patients; SEGA in 5 to 15% of patients	Childhood and adolescence
Pulmonary features		
Key Features	Frequency	Onset symptoms
LAM	Observed in 30 to 40% of the female patients; reaching 80% of affected women after 40 years of age	Adolescence and adult life
Renal features		
Key Features	Frequency	Onset symptoms
Angiomyolipomas ≥2	Observed in 80% of patients	Childhood and adulthood
Secondary features	Frequency	Onset symptoms
Multiple renal cysts	Very varied	Adolescence and adult life
Endocrine features		
Secondary features	Frequency	Onset symptoms
Non-renal hamartomas	Adrenal angiomyolipoma present in 1/4 of the patients	Varied

[A]HM: Hypomelanocytic macules; [B]SEN: Subependimal nodules; [C]SEGA: Giant cell astrocytomas; [D]LAM: Limfangioleiomyomatoses
