# Supplementary material for: The paradox of autophagy in Tuberous Sclerosis Complex
Source: Genet Mol Biol. 2021 Apr 5;44(2):e20200014. doi: 10.1590/1678-4685-GMB-2020-0014 (PMC8022228; doi:10.1590/1678-4685-GMB-2020-0014)
Supplement: Table S2 - [file 1415-4757-GMB-44-2-e20200014-s2.pdf]

Genetics and Molecular Biology
DOI: https://doi.org/ 10.1590/1678-4685-GMB-2020-0014

Supplementary material to: The paradox of autophagy in tuberous sclerosis complex

Table S2 - The main mTOR inhibitors tested in patients with TSC-related manifestations

**Molecule or compound**	**Disease manifestation**	**Objective**	**Sample**	**Treatment methodology**	**Main results**	**[G]Observations**	**Reference**
	[A]AML	Test the effect in reducing the volume of tumors	18 patients with ≥1 renal AML >2 cm in diameter	Oral doses (serum levels 4-8 ng/mL) daily	AML size reduction was 58.8%; reduction persisted after 24 months of treatment	Clinical trial NCT01217125	Cabrera-López *et al*., 2012
	Angiofibromas	Evaluate efficacy and safety	177 patients	1mL of topical formulation containing 1% or 0.1%, applied daily	Appearance were rated better by 65.5% and 81.8% of patients in the 0.1% and 1% groups	Clinical trial NCT01526356	Koenig *et al*., 2018
Rapamycin	Astrocytomas	Evaluate the effect of oral doses on tumor growth	4 patients with SEGAs, 1 with or a pilocytic astrocytoma	Oral doses (serum levels 5–15ng/mL) daily from 2.5 to 20 months	All lesions regressed; interruption of therapy resulted in regrowthing of SEGA in one patient	Case series	Shepherd *et al*., 1991
	[B]HM	Evaluate the efficacy	6 patients with HM	Topic gel at 0.2% was applied twice a day for 12 weeks.	Improvement of HM was significant	Case series	Nabbout *et al*., 1999
	[C]SEGA	Investigate the effect before tumor resection	8-year-old patient with bilateral SEGAs	Oral doses (serum levels 3.3-4.5 ng/mL) daily	Significant reduction of SEGAs size (82.6% and 46.7%) after 3 months	Case report	Franz D *et al*., 2006

Molecule or compound	Disease manifestation	Objective	Sample	Treatment methodology	Main results	[G]Observations	Reference
Everolimus	[C]SEGA	Evaluate efficacy and safety	117 patients	Oral doses (serum levels 5-15 ng/mL) daily	35% of patients had at least 50% reduction in the volume of SEGAs versus none in the placebo group	Clinical trial NCT00789828	Franz *et al*., 2013
	[D]LAM-associated AML	Evaluate efficacy and safety	118 patients with at least one AML	Oral doses at 10 mg	The AML response rate was 42%; occurred reduce of all AML volume	Clinical trial NCT00790400	Bissler *et al*., 2013
	Focal-onset seizures	Evaluate efficacy and safety for treatment-resistant	366 patients	Two concentrations: 3-7 ng/mL (low exposure) and 9-15 ng/mL (high exposure)	The response rate and reduction in seizure frequency was 28.2% and 29.3% for low-exposure and 40% and 39.6% for high-exposure, respectively	Clinical trial NCT01713946	French *et al*., 2016
	[E]RHM	Evaluate efficacy	4 infant cases	Oral doses (serum levels of 5-15 ng/mL) daily	A 50% reduction in HRM sizes was achieved after 1.13 ± 0.33 months of treatment.	Series of cases	Aw *et al*., 2017
	Refractory epilepsy	Evaluate the efficacy and effects on emotional and behavioral symptoms	Four boys and two girls	Oral median dose was 10 mg/day (range 5-20 mg)	All cases experienced very good to moderate response for controlling epileptic seizures; improvement in emotional and behavioral symptoms	Series of cases	Franz and Capal, 2017
	[E]RHM and [C]SEGA	To report data of three neonates treated	Two neonates with RHM and one with SEGA	Oral doses at 0.1 mg daily	Beneficial clinical responses and well-tolerance in all patients	Clinical trial NCT01713946	Goyer; Dahdah; Major, 2015
	Angiofibromas	To test the topical use	A 10-year-old girl	Treatment with 0.4% in petrolatum was applied on the cheeks once daily	After 3 months, the angiofibromas had improved markedly, with even better results after 6 months	Case report	Dil *et al*., 2014

Molecule or compound	Disease manifestation	Objective	Sample	Treatment methodology	Main results	[G]Observations	Reference
Sirolimus	[D]LAM and [A]AML	Evaluate efficacy and safety	16 patients with TSC or sporadic LAM and renal AML	Oral doses (serum levels of 3-10 ng/mL) daily	41 of 48 AMLs of patients were reduced size after treatment	Clinical trial NCT00490789	Davies *et al*., 2011
	Angiofibromas	Evaluate the sustained clinical benefits and safety	10 patients	Topical doses of 0.4% at 3 times a week, for 9 months	Sustained improvement in erythema and in the size and extension of the lesions in all patients	Series of cases	Salido *et al*., 2012
	[E]RHM	Evaluate efficacy and safety	3 infants aged less than 12 months	The oral suspension was 4–10 ng/L	All patients achieved significant reductions in RHM; a complete response was documented in 2 patients	Series of cases	Lucchesi *et al*., 2018
	Kidney [A]AML, [C]SEGA, liver [A]AML, angiofibromas	Evaluate efficacy and safety	36 patients	Target blood level of 3–9 ng/ml for the first 16 weeks; after , a target level was increased to 9–15 ng/ml	The mean decrease in kidney tumor size was 29.9%; was observed regression of SEGAs in 63% cases, of liver AML in 80% cases; improvement in facial angiofibromas in 57%, and stable lung function in women with LAM	Clinical trial NCT00126672	Dabora *et al*., 2011
	[C]SEGA	To summarize their institutional experience for TSC with SEGA, and other syndromes brain tumors	(1) a 9-year-old boy with SEGA; (2) a 13-year-old boy with SEGA and tubers; (3) a 10-year-old boy with headaches and SEGA with significant hydrocephalus	(1) he started with 5 mg daily; (2) he started 6 mg daily; (3) he started with 9 mg daily	(1) 65% SEGA and angiofibromas decreased significantly; (2) headaches improved within 1 month; 60% SEGA decrease after 3 months; (3) 50% SEGA decrease and ventriculomegaly improved, papilledema and headaches had resolved, and adenoma sebaceum lesions had significantly improved	Series of cases	Lam *et al*., 2010
	[A]AML	Evaluate efficacy on the volume of AML	25 patients	Initially, all patients received 0.25 mg /m²; if the target AML had not decreased, the dose was increased 5-10 ng/mL; at the 4-month, if the reduction had not been reached, the dose was increased to 10-15 ng/mL	AMLs regressed somewhat during therapy but tended to increase in volume after the therapy was stopped	Clinical trial NCT00457808	Bissler, *et al*., 2008

Molecule or compound	Disease manifestation	Objective	Sample	Treatment methodology	Main results	[G]Observations	Reference
Rapamycin-tacrolimus	Angiofibroma	Evaluate efficacy and safety	9 patients	0.2% rapamycin in 0.03% tacrolimus ointment as a vehicle	All symptoms, except papule size, began to improve during the 6th week; 1 month after stopping the treatment, redness increased to 60–80% of baseline	Series of Cases	Wataya-Kaneda *et al*., 2011
Metformin	[A]AML and [C]SEGA	Evaluate efficacy	51 patients	The patients received either placebo or metformin	Mean AML volume increase was 9.6% (vs. 25.5% in placebo group); mean SEGA volume increase was 23.3% (vs. 37% in placebo group); there was also a reduction of seizures	Series of cases	Amin *et al*., 2018

[A]AML: Angiomyolipomas; [B]HM: Hypomelanotic macules; [C]SEGA: Subependymal giant cell astrocytomas; [D]LAM: Lymphangiomyolipoma; [E]RHM: Cardiac Rhabdomyomas; [F]RCC: Renal cell carcinoma. [G]Clinical trials numbers are registered under in ClinicalTrials.gov.

References

Amin S, Mallick AA, Edwards H, Lux A, Laugharne M, Likeman M, Khan A and O'Callaghan F (2018) A Randomised, Double-blind, Parallel group, Placebo Controlled Trial of Metformin in Tuberous Sclerosis Complex. Arch Dis Child 103:A4.

Aw F, Goyer I, Raboisson MJ, Boutin C, Major P and Dahdah N (2017) Accelerated Cardiac Rhabdomyoma Regression with Everolimus in Infants with Tuberous Sclerosis Complex. Pediatr Cardiol 38:394-400.

Bissler JJ, McCormack F, Young LR, Elwing JM, Chuck G, Leonard JM, Schmithorst VJ, Laor T, Brody AS, Bean J *et al*. (2008) Sirolimus for angiomyolipoma in tuberous sclerosis complex or lymphangioleiomyomatosis. N Eng J Med 358:140-151.

Bissler JJ, McCormack FX, Young LR, Elwing JM, Chuck G, Leonard JM, Schmithorst VJ, Laor T, Brody AS, Bean J *et al*. (2008) Sirolimus for angiomyolipoma in tuberous sclerosis complex or lymphangioleiomyomatosis. N Engl Journal 358:140-151.

Cabrera-López C, Martí T, Catalá V, Torres F, Mateu S, Ballarín J and Torra R (2012) Assessing the effectiveness of rapamycin on angiomyolipoma in tuberous sclerosis: a two-year trial. Orphanet J Rare Dis 7:87.

Dabora SL, Franz DN, Ashwal S, Sagalowsky A, DiMario Jr FJ, Miles D, Cutler D, Krueger D, Uppot RN, Rabenou R *et al*. (2011) Multicenter phase 2 trial of sirolimus for tuberous sclerosis: kidney angiomyolipomas and other tumors regress and VEGF- D levels decrease. PLoS One 6:e23379.

Davies DM, de Vries PJ, Johnson SR, McCartney DL, Cox JA, Serra AL, Watson PC, Howe CJ, Doyle T, Pointon K *et al*. (2011) Sirolimus therapy for angiomyolipoma in tuberous sclerosis and sporadic lymphangioleiomyomatosis: a phase 2 trial. Clin Cancer Res 17:4071-4081.

Dill PE, De Bernardis G, Weber P and Lösch U (2014) Topical everolimus for facial angiofibromas in the tuberous sclerosis complex. A first case report. Pediatr Neurol 51:109-113.

Franz DN and Capal JK (2017) mTOR inhibitors in the pharmacologic management of tuberous sclerosis complex and their potential role in other rare neurodevelopmental disorders. Orphanet J Rare Dis 12:2-9.

Franz DN, Belousova E, Sparagana S, Bebin EM, Frost M, Kuperman R, Witt O, Kohrman MH, Flamini JR and Wu JY (2013) Efficacy and safety of everolimus for subependymal giant cell astrocytomas associated with tuberous sclerosis complex (EXIST-1): a multicentre, randomised, placebo-controlled phase 3 trial. Lancet 381:125-132.

Franz DN, Leonard J, Tudor C, Chuck G, Care M, Sethuraman G, Dinopoulos A, Thomas G and Crone KR. (2006) Rapamycin causes regression of astrocytomas in tuberous sclerosis complex. Ann Neurol 59:490-498.

French JA, Lawson JA, Yapici Z, Ikeda H, Polster T, Nabbout R, Curatolo P, de Vries PJ, Dlugos DJ, Berkowitz N *et al*. (2016) Adjunctive everolimus therapy for treatment-resistant focal-onset seizures associated with tuberous sclerosis (EXIST-3): a phase 3, randomised, double-blind, placebo-controlled study. Lancet 388:2153-2163.

Goyer I, Dahdah N and Major P (2015) Use of mTOR inhibitor everolimus in three neonates for treatment of tumors associated with tuberous sclerosis complex. Pediatr Neurol 52:450-453.

Koenig MK, Bell CS, Hebert AA, Roberson J, Samuels JA, Slopis JM, Tate P and Northrup H. (2018) Efficacy and safety of topical rapamycin in patients with facial angiofibromas secondary to tuberous sclerosis complex: the treatment randomized clinical trial. JAMA Dermatol 154:773-780.

Lam CH, Bouffet E, Tabori U, Mabbott D, Taylor M and Bartels U (2010) Rapamycin (sirolimus) in tuberous sclerosis associated pediatric central nervous system tumors. Pediatr Blood Cancer 54:476-479.

Lucchesi M, Chiappa E, Giordano F, Mari F, Genitori L and Sardi I (2018) Sirolimus in Infants with Multiple Cardiac Rhabdomyomas Associated with Tuberous Sclerosis Complex. Case Rep Oncol 11:425-430.

Nabbout R, Santos M, Rolland Y, Delalande O, Dulac O and Chiron C (1999) Early diagnosis of subependymal giant cell astrocytoma in children with tuberous sclerosis. J Neurol Neurosurg Psychiatry 66:370-375.

Salido R, Garnacho-Saucedo G, Cuevas-Asencio I, Ruano J, Galán-Gutierrez M, Vélez A and Moreno-Giménez JC (2012) Sustained clinical effectiveness and favorable safety profile of topical sirolimus for tuberous sclerosis - associated facial angiofibroma. J Eur Acad Dermatol Venereol 26:1315-1318.

Shepherd CW, Scheithauer BW, Gomez MR, Altermatt HJ and Katzmann JA (1991) Subependymal giant cell astrocytoma: a clinical, pathological, and flow cytometric study. Neurosurgery 28:864-868.

Wataya-Kaneda M, Tanaka M, Nakamura A, Matsumoto S and Katayama I (2011) A topical combination of rapamycin and tacrolimus for the treatment of angiofibroma due to tuberous sclerosis complex (TSC): a pilot study of nine Japanese patients with TSC of different disease severity. Br J Dermatol 165:912-916.
